# Mechanism of BCDX2-mediated RAD51 nucleation on short ssDNA stretches and fork DNA

**DOI:** 10.1093/nar/gkae770

**Published:** 2024-09-12

**Authors:** Masaki Akita, Paul Girvan, Mario Spirek, Jiri Novacek, David Rueda, Zbynek Prokop, Lumir Krejci

**Affiliations:** Department of Biology and National Centre for Biomolecular Research, Masaryk University, Brno, Czech Republic; Department of Infectious Disease, Faculty of Medicine, Imperial College London, London, UK; Department of Biology and National Centre for Biomolecular Research, Masaryk University, Brno, Czech Republic; Cryo-Electron Microscopy and Tomography Core Facility, Central European Institute of Technology, Brno, Czech Republic; Department of Infectious Disease, Faculty of Medicine, Imperial College London, London, UK; Single Molecule Imaging Group, MRC-London Institute of Medical Sciences, London, UK; Loschmidt Laboratories, Department of Experimental Biology and RECETOX, Faculty of Science, Masaryk University, Brno, Czech Republic; International Clinical Research Center, St Anne's University Hospital, Brno, Czech Republic; Department of Biology and National Centre for Biomolecular Research, Masaryk University, Brno, Czech Republic

## Abstract

Homologous recombination (HR) factors are crucial for DSB repair and processing stalled replication forks. RAD51 paralogs, including RAD51B, RAD51C, RAD51D, XRCC2 and XRCC3, have emerged as essential tumour suppressors, forming two subcomplexes, BCDX2 and CX3. Mutations in these genes are associated with cancer susceptibility and Fanconi anaemia, yet their biochemical activities remain unclear. This study reveals a linear arrangement of BCDX2 subunits compared to the RAD51 ring. BCDX2 shows a strong affinity towards single-stranded DNA (ssDNA) via unique binding mechanism compared to RAD51, and a contribution of DX2 subunits in binding branched DNA substrates. We demonstrate that BCDX2 facilitates RAD51 loading on ssDNA by suppressing the cooperative requirement of RAD51 binding to DNA and stabilizing the filament. Notably, BCDX2 also promotes RAD51 loading on short ssDNA and reversed replication fork substrates. Moreover, while mutants defective in ssDNA binding retain the ability to bind branched DNA substrates, they still facilitate RAD51 loading onto reversed replication forks. Our study provides mechanistic insights into how the BCDX2 complex stimulates the formation of BRCA2-independent RAD51 filaments on short stretches of ssDNA present at ssDNA gaps or stalled replication forks, highlighting its role in genome maintenance and DNA repair.

## Graphical Abstract

**Graphical Abstract:**
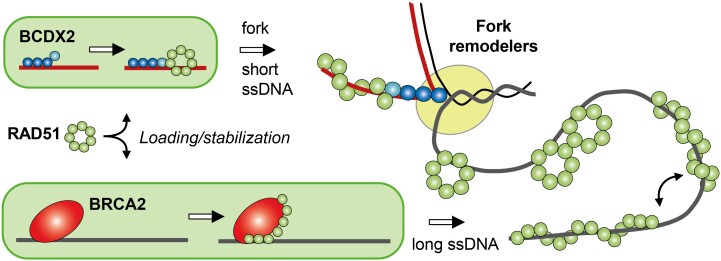

## Introduction

Homologous recombination (HR) is an essential mechanism that ensures the accurate repair of DNA double-strand break (DSB). These breaks can arise spontaneously due to replication fork stalling, collapse, or exposure to DNA-damaging agents. The hallmark of HR is to repair these breaks using homologous donor sequences on homologous chromosomes or sister chromatids, a process essential for maintaining genome integrity. Beside DSB repair, HR also plays a critical role in processing and protecting stalled replication forks. This involves safeguarding newly synthesized DNA strands from degradation by promoting replication fork regression and facilitating the restart of stalled or collapsed replication forks (1). The key player in this process is the recombinase RAD51, which forms nucleoprotein filaments on DNA that protect against nucleolytic degradation and facilitate strand exchange and fork reversal. This multifaceted role highlights the significance of RAD51 in genome maintenance and is further underlined by its mutations associated with cancer and Fanconi anaemia, a rare human disorder linked to defective replication-coupled repair (2–4). The optimal function of the filament relies on many regulatory proteins, including BRCA2 and RAD51 paralogs.

In humans, six members of the RAD51 paralogs family were identified and form three distinct complexes: BCDX2 (RAD51B, RAD51C, RAD51D and XRCC2), CX3 (RAD51C and XRCC3) and SWS (SWSAP1 in complex with SWS1, which is not paralog) (5–12). These complexes garnered attention due to their essential role in maintaining genome stability. Accordingly, cells deficient in any of the paralogs display, similarly to BRCA2-deficient cells, increased sensitivity to DNA-damaging agents and HR- and replication-associated deficiencies (13–16). Genetic alterations of these paralogs across various human cell lines resulted in growth defects, DSB damage sensitivity and impaired HR-mediated DSB repair proficiency. Several mutations in RAD51 paralog genes have been linked to increased breast and ovarian cancer susceptibility (17–21). Moreover, hypomorphic mutations in RAD51C and XRCC2 are associated with Fanconi anaemia (21,22). These findings indicate that BCDX2 is involved in resolving DNA replication stress and maintaining genome stability.

However, the role of RAD51 paralogs is only now emerging. They contain conserved Walker A and B motifs, and most have been demonstrated to possess weak ATPase activity stimulated by ssDNA (11). We have previously identified and characterized a RAD51 paralog complex from *Caenorhabditis elegans*, RFS-1/RIP-1, discovering its critical role in remodeling RAD-51-ssDNA filaments and reducing its dissociation by capping the 5′ filament end to promote strand exchange reaction (23,24). Single-molecule analysis using dimeric RAD51 paralog complexes from *S. cerevisiae* (Rad55/Rad57) and *C. elegans* (RFS-1/RIP-1) revealed cooperative stimulation of RAD51 filament formation on RPA-coated ssDNA (25,26). In addition, Rad55/Rad57 was shown to form complex with Rad51 and stabilize its filaments (27). In contrast to BRCA2, BCDX2 and RAD51 were reported to restrain the progression of replication forks upon stress by promoting fork reversal, thereby limiting ssDNA accumulation and fork breakage (28–30). During preparation of this manuscript, two cryo-electron microscopy studies uncovered the atomic structure of human BCDX2 complex, demonstrating its ability to promote RAD51 filament nucleation and extension, and role of ATPase and DNA binding activities in this process (31,32).

In this study, using various biochemical and biophysical methods, we elucidate the mechanistic role of the BCDX2 complex in promoting RAD51 loading onto short ssDNA stretches and reversed replication fork substrates and quantitatively characterize this process. We demonstrate that BCDX2 exhibits high affinity for ssDNA and branched DNA structures, with significant contributions from RAD51D and XRCC2 subunits. Moreover, while mutants defective in ssDNA binding retain the ability to bind branched DNA substrates, they still facilitate RAD51 loading onto reversed replication forks. Our results offer a novel mechanistic insight into RAD51 loading by the RAD51 paralog complex. We also discuss how the BCDX2 complex stimulates the formation and stabilizes BRCA2-independent RAD51 filaments on short ssDNA segments, highlighting its difference to BRCA2 and underlying its significance in genome maintenance and DNA repair.

## Materials and methods

### Baculovirus expression vectors

To facilitate the expression of human RAD51 paralogs (*RAD51B*, *RAD51C*, *RAD51D* and *XRCC2*) in insect cells, we first optimized their codons for enhanced expression and synthetized the respective sequences. Subsequently, we cloned N-terminally Strep II tagged RAD51B, RAD51C, RAD51D and N-terminally 10xHis tagged XRCC2 into a pGBdest (pGBdest BCDX2) for baculovirus expression. To specifically express the DX2 complex, we cloned RAD51D and N-terminally 10xHis tagged XRCC2 into another pGBdest vector (pGBdest DX2). For the baculoviral expression of the BC complex, the pGBdest BCDX2 was first digested with NcoI and FspI to excise both *RAD51D* and *XRCC2* genes. Subsequently, following blunting of the DNA ends, we generated the pGBdest BC vector through self-ligation.

### Protein expression and purification

For preparation of corresponding bacmids, all vectors were transfected into MAX Efficiency DH10Bac Competent Cells (Thermo Fisher Scientific), and recombinant bacmid DNA was isolated according to the manufacturer's procedures. Primary and secondary baculoviruses were generated following the protocols outlined for the Bac-to-bac system (Life Technologies) using Sf9 insect cells (Thermo Fisher Scientific). High Five cells (500 ml, 2 × 106 cells/ml) were grown in flasks in Express Fivetm SFM media (Gibco) together with 1/100 volume of corresponding virus. After 120 hr of incubation at 28°C, the cells were pelleted at 1000 rpm at 4°C and washed once with cold 1x PBS buffer (137 mM NaCl, 2.7 mM KCl, 10 mM Na_2_HPO_4_ and 1.8 mM KH_2_PO_4_, adjusted pH to 7.4 with NaOH). The cell pellets were frozen and kept at -80°C. For purification, the cells were resuspended in 50 ml of buffer A (20 mM KH_2_PO_4_ (pH 7.5), 0.3 M KCl, 10% Glycerol, 0.5 mM EDTA, 0.1% NP-40, 1 mM DTT, 2 mM MgCl_2_ and 1 mM ATP) with cOmplete(TM), Mini, EDTA-free Protease inhibitor Cocktail (Roche) (1 tablet per 25 ml buffer). The cells were lysed by sonication on ice, performing 5 cycles of 1 min each using a Hielscher UP200S Ultrasonic Processor (duty cycle 0.3, Amplitude 70%). The lysate was cleared in Avanti J-26S XPI Centrifuge (Beckman Coulter) with a JA-30.50Ti rotor at 19 000 rpm for 60 min at 4°C.

For BCDX2, the supernatant was bound to 2 ml bed volume of Strep-Tactin beads (IBA Lifesciences) by rotating at 4°C for 2 h, then applied to Econo-Column (BIO-RAD). All subsequent steps were carried out at 4°C. The flowthrough fraction was discarded, and the beads were washed with 50 ml buffer A. The protein was eluted twice with 8 ml of buffer A containing 5 mM desthiobiotin (IBA Lifesciences). Imidazole was added to the eluted fraction to a final concentration of 50 mM and applied to 2 ml bed volume of Ni-NTA agarose affinity gel (Qiagen 30210), which had been pre-washed with buffer A containing 50 mM Imidazole. The protein was bound to the beads by rotating overnight, then applied to Econo-Column. The flowthrough was discarded, and the beads were washed with 50 ml buffer A containing 50 mM imidazole. The protein was eluted with buffer A containing 500 mM imidazole, and 0.5 ml fractions were collected. The peak fractions were pooled and dialyzed against 1 l of buffer B (20 mM KH_2_PO_4_ (pH 7.5), 10% glycerol, 0.5 mM EDTA, 0.1% NP-40, 2 mM MgCl_2_ and 1 mM DTT) containing 300 mM KCl using Spectra/Por 1 Dialysis Membranes MWCO: 6000–8000 (SPECTRUMLABS. COM) for 1 hr. Aliquots were stored at −80°C.

For BC complex, the supernatant from cell lysate after centrifugation was bound to 2 ml bed volume of Strep-Tactin beads, which had been pre-washed with buffer A by rotating at 4°C for 2 h, then applied to an Econo-Column. The flowthrough was discarded, and the beads were washed with 50 ml buffer A. The protein was eluted twice with 8 ml of buffer A containing 5 mM desthiobiotin. The complex was diluted with 40 ml of buffer B to reduce salt concentration to 50 mM KCl. The protein was bound to 1 ml Mono Q 5/50 GL column (GE Healthcare) at 0.5 ml/min using Äkta Explorer HPLC system and washed with 10 ml buffer B containing 50 mM KCl. The BC complex was eluted with 10 ml gradient of 50–500 mM KCl in buffer B, and 0.5 ml fractions were collected. The peak fractions were pooled and then dialysed against buffer B containing 300 mM KCl for 1 hr. Aliquots were stored at -80°C.

For DX2 complex, the supernatant from cell lysate after centrifugation was bound to a 2 ml bed volume of Ni-NTA agarose affinity gel, which had been pre-washed with buffer A containing 50 mM imidazole. The protein was bound to the beads by rotating for 2 hr, then applied to an Econo-Column. The flowthrough was discarded, and the beads were washed with 50 ml buffer A containing 50 mM imidazole. The protein was eluted with buffer A containing 500 mM imidazole, and 0.5 ml fractions were collected. The peak fractions were mixed at 1:2 ratio with buffer C (25 mM Tris–HCl (pH 7.4), 10% Glycerol, 0.5 mM EDTA, 2 mM MgCl_2_ and 0.1% NP-40). The protein was loaded onto a 1 ml Ceramic Hydroxyapatite Type I column (BIO-RAD) equilibrated with buffer C containing 100 mM KCl at a flow rate 0.5 ml/min using Äkta Explorer HPLC system. The column was washed with 10 ml buffer C containing 100 mM KCl and the protein eluted with a 10 ml gradient of 0–500 mM KH_2_PO_4_ in buffer C, and 0.5 ml fractions were collected. The peak fractions were pooled and dialysed against buffer B containing 300 mM KCl for 1 hr. Aliquots were stored at −80°C.

RAD51 was overexpressed in *E. coli* and purified as described previously (33). After purification, RAD51 was pooled and dialyzed against buffer C containing 300 mM KCl for 1 h. Aliquots were stored at -80°C. DX2 mutants, namely D(R266A)X2 and DX2(R159A), were provided by Patrick Sung lab.

For size exclusion chromatography, the dialyzed BCDX2 protein sample was loaded on Superdex200 Increase 5/150GL column (Cytiva) equilibrated in buffer A. The sample was eluted with flow rate 0.1 ml/min and fractionated with 0.05 ml/tube. RAD51 protein pre-incubated in 1 mM ATP and 5 mM MgCl_2_ was loaded on Superdex200 Increase 10/300GL column in the presence of 50 mM KCL, protein was eluted with flow rate 0.15 ml/min and fractionated with 0.5 ml/tube. Protein samples were resolved on SDS-PAGE. The gels were scanned using Amersham Typhoon (Cytiva), and peak fraction were assessed using ImageQuant TL (Cytiva). RAD51 was detected by Western-blot analysis using rabbit anti-RAD51 (BioAcademia). Protein complex of human miniBRCA2 and DSS1 was purified from insect cells according to (34).

### Mass photometry

Mass photometry measurements were performed on a Refeyn Two MP instrument. Calibration was carried out using BSA and thyroglobulin. Sample carrier slides (Refeyn) were used with six well gaskets (Grace-Bio-Labs, CultureWell 50–3mm) in which samples were placed. BCDX2 was diluted to 20 nM in 50 mM Tris–HCl, pH 7.5, 50 mM NaCl, 5 mM MgCl_2_, 1 mM CaCl_2_, 2 mM ATP prior to measurement. Data was acquired using the AcquireMP software using default settings and then analysed with DiscoverMP to determine the mass of each detected particle, which was then plotted as a histogram.

### Electron microscopy analysis

Protein preparation: Freshly eluted BCDX2 complex from Ni-NTA agarose affinity gel was loaded onto Superdex200 Increase 10/300 GL column equilibrated in buffer D (20 mM KH_2_PO_4_ (pH 7.5), 10% Glycerol, 0.5 mM EDTA, 0.1% NP-40 2 mM MgCl_2_ and 1 mM DTT) containing 300 mM KCl. Peak Fractions (350 nM or 0.004 mg/ml) were pooled.

Sample preparation: The purified BCDX2 complex (0.02 mg/ml, 3.5 μl) was incubated in buffer M (20 mM KH_2_PO_4_ (pH 7.5), 10% Glycerol, 0.5 mM EDTA, 0.1% NP-40, 2 mM MgCl_2_ and 1 mM DTT) containing 300 mM KCl, 1 mM ATP or 5 nM ssDNA or RVF1 (Supplementary Table S1) for 10 min at 37°C and then applied to the freshly glow discharged TEM grids (Cu, 400mesh) coated with 12 nm thick homemade continuous carbon film. The sample was incubated for 30 s on the grid followed by staining in two drops of nanoW (Nanoprobes). Alternatively, the BCDX2 complex was incubated with 5-fold molar access of 1.8 nm Ni-NTA-Nanogold (Nanoprobes) and stained using same procedure as described above. Data acquisition: Negative stain electron microscopy micrographs were collected using Talos TF200C transmission electron microscope (ThermoScientific) operated at 200 kV using SerialEM software (PMID:16182563). The data were collected on the CetaD camera (ThermoScientific) at the calibrated pixel size of 1.59 Å/pixel, overall dose of 40 e/Å (2), and with the defocus ranging 2–4 μm. Data processing: The micrographs (942 micrographs) were imported into CryoSparc (PMID:28165473) and processed according to the single particle analysis workflow. Templates for automated particle picking were generated from manual picking of 20 micrographs. The false positive picks and corrupted particles were removed from the dataset through multiple rounds of reference-free 2D classification. 3D classification was carried out through the Ab initio job in CryoSparc. The initial model (generated from the Ab initio job) was then refined using homogeneous refinement. The Alphafold2 (PMID: 34265844) was used for prediction of the BCDX2 structural arrangement, and the coordinates were subsequently fit into the negative-stain EM model as a rigid body in Chimera (PMID:15264254). The RAD51 protein and ssDNA-filaments were analysed in buffer containing 50 mM Tris–HCl pH 7.5, 50 mM KCl, 10 mM MgCl2, and 1 mM ATP. RAD51 protein (5.4 μM) was mixed with 16.2 μM phi X 174 (concentration in nucleotides) and incubated for 10 min or 6 h at 37°C. The sample (4 uL) was deposited on glow-discharged grids coated with 12 nm layer of homemade continuous carbon and stained with uranyl acetate. Data were collected on transmission electron microscope Tecnai F20 (FEI, Eindhoven) operating at 200 keV with a nominal magnification of 50,000x resulting in pixel size of 2.22 Å/px. The data were acquired on CCD camera (FEI Eagle) under low-dose conditions (∼25 e^−^/Å^2^ s) and with the underfocus in the range of 2.5–4.5 μm.

### Electrophoretic mobility shift assay (EMSA)

Proteins were diluted from concentrated stocks into buffer B containing 300 mM KCl, which was also used in the no protein controls. Proteins were mixed at 37°C with a master mix containing 20 nM FITC-labeled DNA substrates (see Supplementary Table S1 for individual substrates), 20 mM Tris–HCl (pH 7.5), 2 mM MgCl_2_ and 2 mM ATP, and incubated for 10 min in 10 μl reaction volume, before crosslinking with 5 μl of 0.5% glutaraldehyde for 5 min. Reactions were resolved on 0.9% agarose gels in TAE (90 V, 60 min), gels were scanned using Amersham Typhoon (Cytiva), and percentage of DNA binding was assessed using ImageQuant TL (Cytiva), free/unbound DNA was quantified. *P* values were calculated using Microsoft Excel software.

### Stopped-flow analysis

The stopped-flow experiments were done as described previously. (35) Briefly, all reactions were performed in SF buffer, consisting of 25 mM Tri–HCl (pH 7.5), 5 mM MgCl_2_, 50 mM KCl, 2 mM ATP, 0.5 mM EDTA and 10% glycerol. The reactions contained 30 nM Cy3-labeled oligonucleotide (Cy3-43mer), with the fluorophore conjugated to either the 5′ or 3′ end (Supplementary Table S1) and performed at 37°C. For titration experiments involving RAD51 filaments or BCDX2, we employed the 43mer and used non-saturating concentrations of RAD51 (0–2000 nM) or BCDX2 (0–400 nM), to ensure that not all ssDNA molecules were coated by RAD51 filaments. Fluorescence measurements for most experiments were collected according to the following protocol: (1) every 0.00005 s from 0 to 0.05 s; (2) every 0.0005 s from 0.05 to 0.56 s; (3) every 0.02 s from 0.56 to 60.54 s using BioLogic Bio-kine32 software. Kinetic data analysis and statistics are described in Supplementary Data section.

### Pulldown assay

To detect the interaction between BCDX2 and RAD51, we mixed 5 μg of purified BCDX2 (containing Strep-tagged RAD51B) and 5 μg of purified RAD51 together with 4 μl of Strep-Tactin beads. The reaction was prepared in total volume of 200 μl of PD buffer (25 mM Tris–HCl (pH 7.5), 0.5 mM DTT, 100 mM KCl, 0.1 μg/ml BSA, 10% Glycerol, 0.01% NP-40 and 2 mM ATP), and incubated on ice for 1 hr. Subsequently, the beads were then washed 10 times with 1 ml of wash buffer I (25 mM Tris–HCl (pH 7.5), 0.5 mM DTT, 100 mM KCl, 3 mM EDTA, 0.01% NP-40 and 2 mM ATP), to remove any non-specifically bound proteins. The bound proteins were subsequently eluted using 60 μl of wash buffer I supplemented with 5 mM desthiobiotin for 15 min on ice. The input and bead fractions were resolved in SDS-PAGE, followed by western blotting using anti-XRCC2 antibody (Abcam ab190752, 1:2000 dilution) to detect XRCC2 and anti-RAD51 antibody (Bio Academia 70-001, 1:5000 dilution) to detect RAD51. Western blot images were captured by LAS-4000 (Fujifilm) and subsequently analysed by Fujifilm MultiGauge software.

### Biolayer-interferometry assay (BLI)

BLI experiments were performed using a single–channel BLItz instrument in Advanced Kinetics mode (ForteBio) at room temperature and with continuous shaking at 2.200 rpm. Prior to the measurements, streptavidin biosensors (SAX, ForteBio, Cat No. 18- 0037) were pre–hydrated by incubation with BLI buffer (50 mM Tris–HCl (pH 7.5), 2 mM ATP, 50 mM KCl and 0.05% Tween 20) for 10 min. Subsequently, the biosensor was loaded with 15 nM biotinylated dT ssDNA (Supplementary Table S1) for 120 seconds and washed with BLI buffer containing 5 mM MgCl_2_. Next, a solution containing RAD51 (0.5 μM), BCDX2 (0.05 or 0.1 μM), or mixture of both proteins was applied to the biosensor in BLI buffer containing 5 mM MgCl_2_, and association was monitored for 240 seconds. The binding affinity of the proteins to DNA was determined by measuring the increase in the thickness of the biomolecule layer in nanometers (nm). To monitor the dissociation of RAD51 filaments, 100-fold of unlabelled ssDNA in BLI buffer was mixed with RAD51 filaments in dissociation phase. To inhibit ATPase activity of BCDX2, 10 μM BCDX2 was preincubated with 100 μM sodium vanadate for 10 min at 25°C.

### Single-molecule FRET (smFRET) microscope setup

Single-molecule FRET measurements were performed on a homebuilt prism-TIRF (Total Internal Reflection Fluorescence) microscope as described. (36) Fluorophores were excited by a 532 nm laser (Stradus, Vortran) and fluorescence was collected through a 1.2 NA, 60× water objective (Olympus) and filtered through a long-pass filter (ET542lp, Chroma). Donor and acceptor emission was spectrally separated using an OptoSplit II (Cairn Research) with ET585/65M and ET700/75M (Chroma) bandpass filters, respectively. The donor and acceptor images were then projected side-by-side onto an EMCCD camera (Andor iXon Ultra 897). Data was collected as raw movies using a custom LabVIEW script at 100 ms time resolution.

Single molecule fluorescence spots from the raw movies were localized using custom IDL scripts and converted into raw fluorescence trajectories. Raw fluorescence trajectories were corrected for bleed-through of the donor fluorescence into the acceptor channel. Apparent FRET efficiencies were calculated as the ratio of acceptor intensity divided by the sum of the donor and acceptor intensities. Acquisition details and analysis are described in Supplementary Data section.

## Results

### BCDX2 complex displays a linear arrangement and exhibits stronger binding affinity towards branched DNA containing single-stranded DNA

To characterize the role of RAD51 paralogs, we successfully co-expressed and purified human RAD51 paralog complexes (BCDX2, BC and DX2) to near homogeneity from insect cells (Supplementary Figure S1A). The presence of all subunits was further verified by mass spectroscopic analysis (Supplementary Figure S1B). Next, we performed gel filtration to test the assembly of the BCDX2 complex. The elution profile indicates that BCDX2 forms a complex of approximately 150 kDa (Supplementary Figure S1C), similarly to histogram observed using mass photometry (Supplementary Figure S1D), corresponding to a heterotetramer. Further structural assessment was carried out by electron microscopy (EM). EM visualization and reconstruction of the BCDX2 complex in the presence of ATP and MgCl_2_ revealed a linear arrangement of subunits, in agreement with structural prediction using AlphaFold (Supplementary Figure S2A–C). This contrasts to oligomeric and ring-like structures of RAD51 protein seen by gel filtration and EM, respectively (Supplementary Figure S1E and F). The fitting analysis indicated an apparent stoichiometry of BCDX2 as 1:1:1:1, with RAD51B and XRCC2 subunits located at the ends of the BCDX2 complex. Furthermore, EM imaging of Ni NTA-nanogold particles bound to N-terminally His-tagged XRCC2 further confirmed its position at the end of the complex (Supplementary Figure S2D). These findings agree with recently reported BCDX2 structures (31,32).

**Figure 1.**
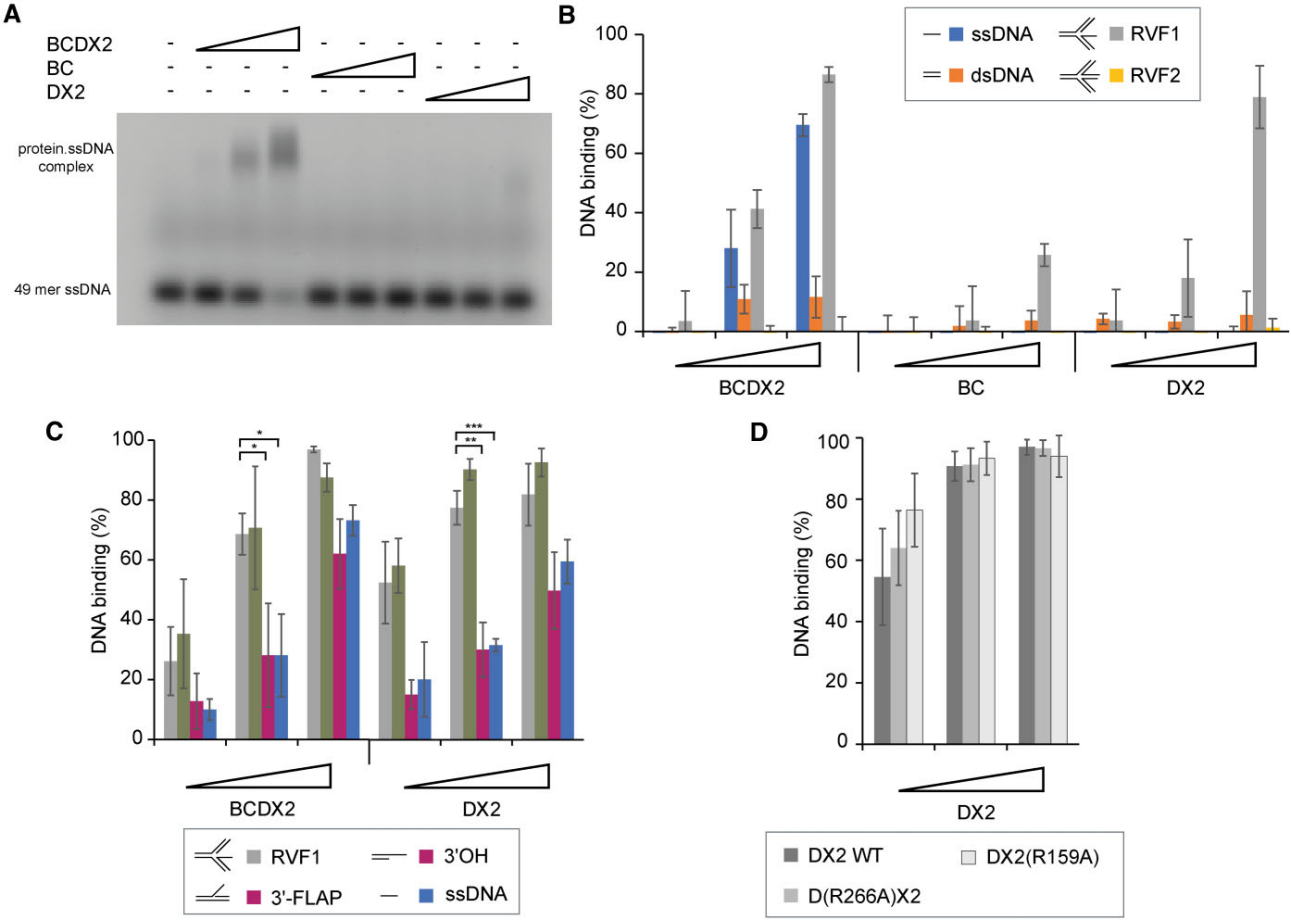
BCDX2 binds ssDNA, with DX2 subcomplex facilitating recognition of branched DNA (**A**) EMSA of BCDX2, BC, and DX2 complexes (100, 200, and 400 nM) binding to FITC-labelled ssDNA (49mer, 20 nM) for 10 min. Following the incubation, protein-DNA complexes were crosslinked and resolved in an agarose gel. (**B**) Percentage of DNA binding by BCDX2, BC and DX2 complexes (100, 200 and 400 nM) to various DNA substrates (20 nM), including ssDNA, dsDNA, reversed replication fork containing ssDNA overhang (RVF1) and fully reversed fork-like substrate (RVF2). *n* = 3 independent experiments; data are means s.d. (**C**) Percentage of DNA binding by BCDX2 and DX2 complexes (100, 200 and 400 nM) to various DNA substrates (20 nM), including ssDNA (27mer), 3′ overhang, 3′flap, and reversed replication fork containing ssDNA overhang (RVF1). n = 3 independent experiments; data are means s.d. *P*-values are obtained by Student's *t*-test (two-tailed): * *P* ≤ 0.05; ** *P* ≤ 0.01, *** *P* ≤ 0.001. (**D**) Percentage of DNA binding by DX2 wild type complex or its mutant variants, namely D(R266A)X2 and DX2(R159A) (100, 200 and 400 nM), to reversed replication fork containing ssDNA overhang (RVF1) (20 nM), *n* = 3 independent experiments; data are means s.d.

Subsequently, we tested the DNA binding specificity of BCDX2 using electrophoretic mobility shift assay (EMSA). Our results showed that BCDX2 preferentially binds to ssDNA with little or no affinity to dsDNA and fully reverse fork-like substrate (RVF2), while BC or DX2 subcomplexes showed no significant binding to either DNA form (Figure 1A and B, and Supplementary Figure S3A). Intriguingly, we observed that BCDX2 and DX2 complexes displayed strong binding to a reverse fork substrate containing protruding ssDNA (RVF1), indicating that branch DNA structures with ssDNA region are more favourable for DNA binding. This finding was further supported by stronger binding to Y-form and flap DNA substrates compared to ssDNA, overhang, gapped DNA, and fork with no protruding ssDNA (Figure 1C and Supplementary Figure S3A–D). Analysis of DNA binding affinities between BCDX2 and DX2 suggests that while all subunits are required for ssDNA binding, RAD51D and XRCC2 subunits significantly contribute to the structure-selective DNA binding activity of the complex (Figure 1B and C, and Supplementary Figure S3A-C). Furthermore, we tested two mutants within DX2 complex, namely D(R266A)X2 and DX2(R159A), which were recently reported to strongly or moderately affect ssDNA binding (32) (Supplementary Figure S4A). Interestingly, these mutants did not significantly affect the binding to branched DNA (Figure 1D and Supplementary Figure S4B), indicating the presence of two separate DNA binding domains or mechanisms withing the DX2 complex. This finding aligns with the observed conformational change of BCDX2 upon binding to RVF1 by EM, in contrast to ssDNA binding (Supplementary Figure S2C). In summary, our data show linear and equimolar stoichiometry of the BCDX2 complex arrangement which has high affinity for ssDNA and branched DNA structures containing ssDNA overhang. In addition, DX2 significantly contributes to this binding, which appears to possess two distinct DNA binding domains or mechanisms.

### Different binding mechanisms of ssDNA interaction with BCDX2 and RAD51

**Figure 2.**
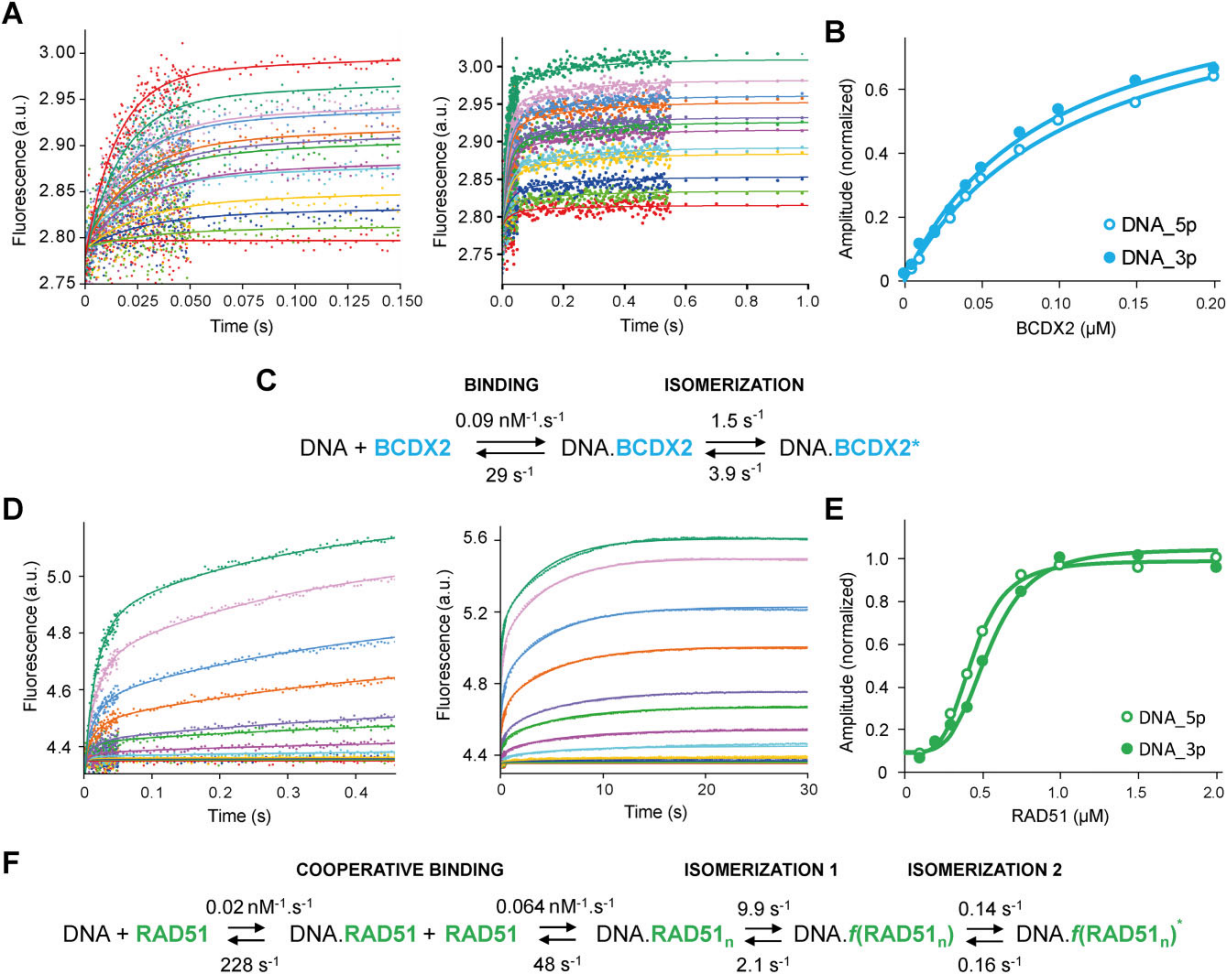
Kinetics and mechanism of DNA interaction with RAD51 and BCDX2. (**A**) Stopped-flow fluorescence data (excitation 545 nm, emission > 550 nm) recorded upon mixing of 30 nM Cy3-labelled DNA with BCDX2 (0, 20, 40, 60, 80, 100, 120, 140, 160, 180, 200, 300 and 400 nM). The initial rapid binding phase (0 to 0.15 s; left) is followed by a slower isomerization step (up to 1 s; right). (**B**) The hyperbolic relationship between the equilibrium amplitude and BCDX2 concentration, indicating a simple binding interaction of BCDX2 and DNA. The open or filled circles represent a data for 5′ or 3′end labelled DNA. (**C**) The kinetic model of the simultaneous action of BCDX2. The rate constants for individual steps were determined by global numerical analysis. The experiment was performed in two independent repetitions. Complete data and results of kinetic analyses are summarized in Supplementary Figure S5. (**D**) Stopped-flow fluorescence data recorded upon mixing 30 nM Cy3-labelled DNA with RAD51 (0, 25, 50, 100, 200, 300, 400, 500, 750, 1000, 1500 and 2000 nM). The initial binding phase (0 to 0.5 s; left) is followed by a slow filament formation (up to 30s; right). (**E**) The sigmoidal relationship between the equilibrium amplitude on RAD51 concentration, indicating a cooperative mode of RAD51 binding to DNA. The open or filled circles represent a data for 5′ or 3′end labelled DNA. (**F**) The kinetic model illustrating the simultaneous action of RAD51. The rate constants for individual steps were determined by global numerical analysis. The experiment was performed in two independent repetitions. Complete data and results of kinetic analyses are summarized in Supplementary Figure S6.

To further elucidate the specific binding mechanism of the BCDX2 (Figure 2A–C) complex and RAD51 (Figure 2D–F) with ssDNA a transient kinetics approach was employed. Using the stopped-flow assay, we monitored the kinetic phases of changes in Cy3 fluorescence as a proxy of BCDX2 and RAD51 binding to ssDNA (33). In these experiments, a constant concentration of 3‘-Cy3 labeled dT43 was rapidly mixed with various concentrations of BCDX2 or RAD51 proteins. The recorded association transients for BCDX2 showed a rapid binding that reached apparent equilibrium within just one second (Figure 2A). Notably, the kinetic data exhibit a double-exponential character, indicating two predominant steps: the initial binding of BCDX2 to DNA followed by a subsequent isomerization associated with structural change in this complex (Figure 2A–C, and Supplementary Figure S5). Conversely, the kinetic data recorded for the interaction between DNA and RAD51 revealed a complex behaviour, with equilibrium being reached approximately after 30 seconds, featuring four distinct exponential phases (Supplementary Figure S6). We began the kinetic analysis using conventional analytical fitting to develop an initial kinetic model and obtain preliminary estimates of some of the rate and equilibrium constants (see Supporting Information Text ‘Conventional Analytical Fitting of Kinetic Data’ and Supplementary Figure S7). While this approach provides valuable mechanistic insights, particularly through the analysis of the concentration dependence of rates and amplitudes, it has limitations in providing precise parameter estimates due to simplifying approximations and error accumulation during multistep fitting. To address these limitations, we performed a global analysis of the kinetic data. This involved a detailed kinetic analysis, employing a numerical integration method, allowed us to formulate a comprehensive interaction model expanding our current view (33). This model consists of a two-step binding process of RAD51 to DNA, characterized by cooperative behaviour, followed by two subsequent isomerization steps (Figure 2F). The weak initial interaction of RAD51 with DNA is the main barrier in the reaction pathway. However, once the initial complex is formed, it facilitates binding of additional RAD51 molecules to form a more stable DNA.RAD51 complex. It is important to mention that the cooperative mechanism becomes effective only at higher concentrations of RAD51 above 200 nM (Figure 2E).

The cooperative binding of RAD51 is subsequently followed by an isomerization step, associated with the formation of the RAD51.DNA nucleoprotein filaments. The final step of RAD51 filament assembly is characterized by another isomerization, resulting in dynamic transitions of the filament (Figure 2F). The higher value for the reverse rate relative to the forward rate indicates possible structural flexibility and dynamic fluctuations between these two filament forms. Interestingly, re-evaluation of binding of RAD51 Walker A mutants to ssDNA, namely K133R and K133A (33), shows high similarity to wild type protein except for marked reduction in cooperativity (Supplementary Figure S7A-D), indicating a role of ATP in the cooperative binding of RAD51. An analysis of individual interactions showed that BCDX2 loads to DNA at higher velocity with almost two orders of magnitude higher affinity, signifying the increased stability of the bound complex (equilibrium association constant *K_a_ = 1/K_d_ = k_1_/k_-1_* is 3.1 μM^−1^ for DNA.BCDX2 and 0.09 μM^−1^ DNA.RAD51). Additionally, our comparison of binding interactions using DNA labeled at different termini (3′ and 5′ ends) shows that neither RAD51 nor BCDX2 exhibited a significant preference for either end (Figure 2B and E). Collectively, our comprehensive mechanistic characterization of ssDNA binding has unveiled different mechanisms for BCDX2 and RAD51, shedding light on the intriguing diversity in the strategies employed by these orthologues to interact with ssDNA.

### BCDX2 physically interacts with RAD51 and facilitates binding of RAD51 on ssDNA.

**Figure 3.**
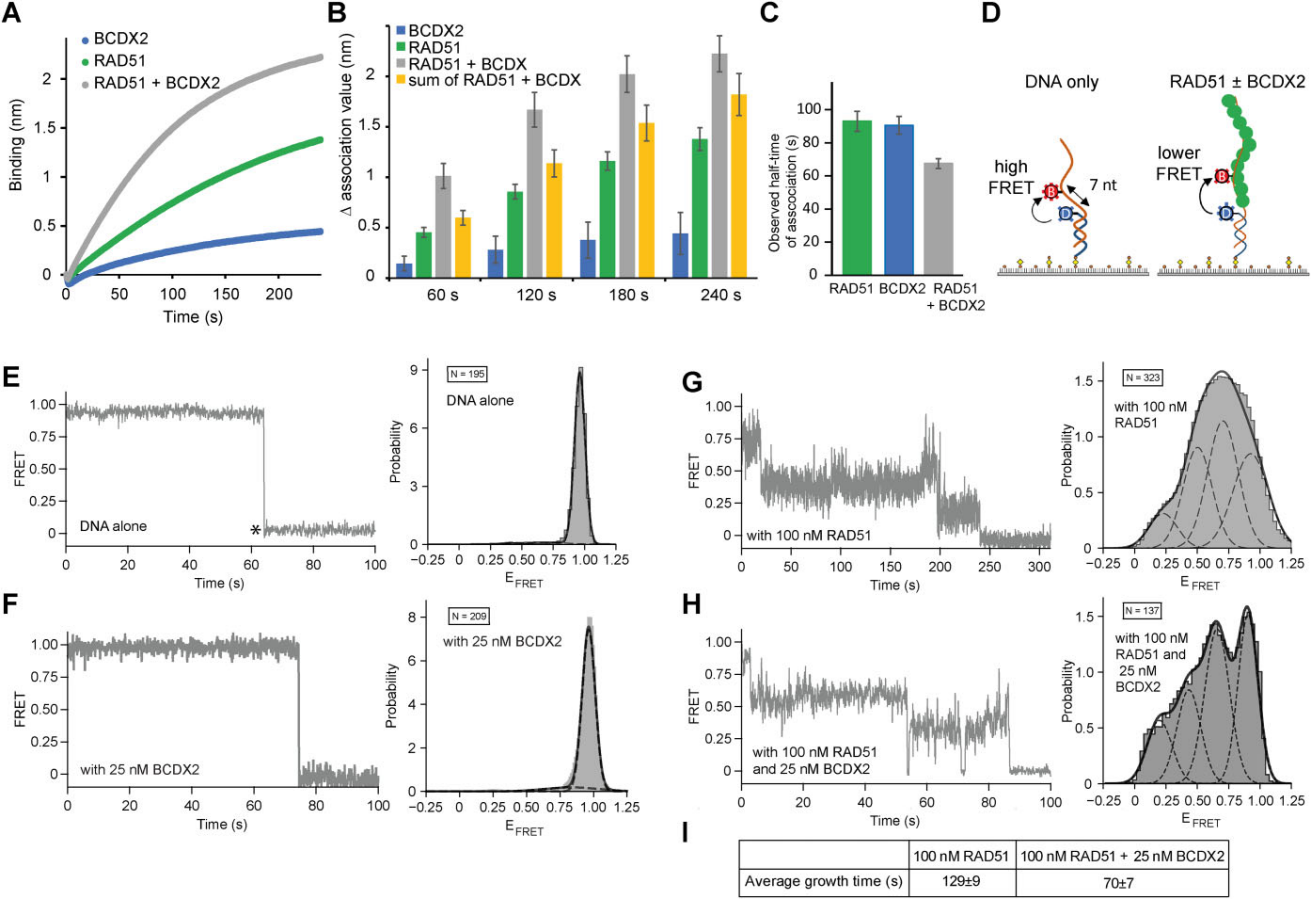
BCDX2 facilitates loading of RAD51 on ssDNA. (**A**) Bio-layer interferometry (BLI) sensorgrams obtained using 15 nM 5′-biotinylated ssDNA conjugated to streptavidin-loaded biosensors. A solution of RAD51 (0.5 μM), BCDX2 (0.05 μM), or their mixture was loaded onto the biosensor in presence of ATP/Mg and association was monitored. (**B**) Graph depicting changes in binding amplitudes of RAD51, BCDX2, their mixture or sum of values for RAD51 and BCDX2 alone at various time points. The values for association phase were normalized to the starting point (time point = 0 s). *n* = 3 independent experiments; data are means s.d. (**C**) Graph of observed half-time of association in the binding of RAD51, BCDX2, or their mixture. *n* = 3 independent experiments; data are means s.d. (**D**) Schematic diagram of the smFRET experiment. Biotinylated ss/ds-DNA constructs immobilized on a streptavidin-coated PEG surface and labeled with FRET donor (blue, Cy3) and acceptor (red, Cy5) separated by 7-nt report on the formation of RAD51 filament formation as a stepwise decrease from high FRET (DNA only). (**E**) smFRET trajectory (left) and FRET histogram (right, *N* = 195 trajectories) of DNA alone. DNA alone exhibits static high (0.96) until dye photobleaching (asterisk). Experimental histogram (grey) matches the FRET histogram (black line) derived from applying a global HMM to the individual trajectories. (**F**) smFRET trajectory (left) and FRET histogram (right, *N* = 209 trajectories) of BCDX2 alone. Experimental histogram (grey) matches the FRET histogram (black line) derived from applying a global HMM to the individual trajectories. (**G**) smFRET trajectory (left) and FRET histogram (right, *N* = 323 trajectories) in the presence of 100 nM RAD51. smFRET trajectory shows the expected stepwise decrease in FRET indicating RAD51 filament formation. FRET histogram (grey) reveals the appearance of several lower FRET states upon RAD51 filament formation. A global HMM analysis of the individual trajectories yields the individual (dashed) and sum (solid) FRET histograms, matching the experimental histogram. (**H**) Characteristic smFRET trajectory (left) and FRET histogram (right, *N* = 137 trajectories) in the presence of 100 nM RAD51 and 25 nM BCDX2. smFRET trajectory shows a faster stepwise decrease in FRET and RAD51 filament formation. FRET histogram (grey) reveals the appearance of similar lower FRET states in the presence of BCDX2. A global HMM analysis of the individual trajectories identifies similar individual FRET states in the presence and absence of BCDX2 (compare F and G). (**I**) The table showing quantifications of growth rates from smFRET analysis shown in panels G and H.

To address the effect of BCDX2 on RAD51, we first confirmed their previously identified direct physical interaction (31,32,37), through a pulldown experiment using immobilized BCDX2 on Strep-Tactin beads and RAD51 (Supplementary Figure S8A). To investigate the functional implications of the interaction, we initially assessed the influence of BCDX2 on RAD51 association on ssDNA using a Bio-Layer Interferometry (BLI) assay. Employing non-saturating concentrations of RAD51 (0.5 μM), BCDX2 (0.1 μM), or their combination, we loaded them onto dT43 ssDNA-conjugated biosensor in the presence of ATP and Mg2 + to monitor protein association dynamics (Figure 3A). Examination of the association phases revealed an increase in RAD51 association and decrease in half-time of the reaction in the presence of BCDX2 (Figure 3B and C), suggesting that BCDX2 facilitates the RAD51’s binding to ssDNA. Notably, the stimulatory effect of BCDX2 was most pronounced at early time points and exceeded the expected additive effect of BCDX2 and RAD51 alone (Figure 3B). In summary, we have confirmed direct protein interaction between RAD51 and BCDX2 and demonstrated the ability of the BCDX2 complex to facilitate association of RAD51 with ssDNA.

### Single-molecule FRET reveals enhanced RAD51 filament loading and elongation by BCDX2

To gain deeper insight into the role of BCDX2 on the assembly of RAD51 filament, we performed single-molecule FRET (smFRET) experiments (23). Filament properties were monitored by measuring FRET efficiency between a Cy3 donor and a Cy5 acceptor fluorophore, strategically separated by seven nucleotides within surface-immobilized ssDNA constructs (Figure 3D). The FRET trajectories between Cy3 and Cy5 exhibit a sharp drop to zero FRET upon acceptor photobleaching (Figure 3E). To monitor the effect of BCDX2 we selected non-saturating condition of RAD51 (100 nM), which exhibited a discernible FRET decrease relative to DNA or BCDX2 alone (Figure 3E–G). With the introduction of BCDX2 in combination with RAD51 (100 nM), an even more pronounced FRET decrease was observed (Figure 3H). These results indicated an accelerated filament dynamics in the presence of BCDX2.

In depth analysis of the smFRET data facilitated the determination of the average time required for a molecule to transition from a high FRET state (no protein) to the lowest FRET state (RAD51 filament). Notable, the mean time for RAD51 alone to achieve this transition was calculated at 129 ± 9 s. However, upon the addition of BCDX2, this mean growth time notably decreases to 70 ± 7 s (Figure 3I). This compelling finding indicated an approximately 80% increase in filament growth rate in the presence of BCDX2. Taken together, our single-molecule FRET analysis strongly corroborates the pivotal role of BCDX2 in RAD51 filament loading on ssDNA and its elongation.

### Unveiling the model of RAD51 filament loading by BCDX2

**Figure 4.**
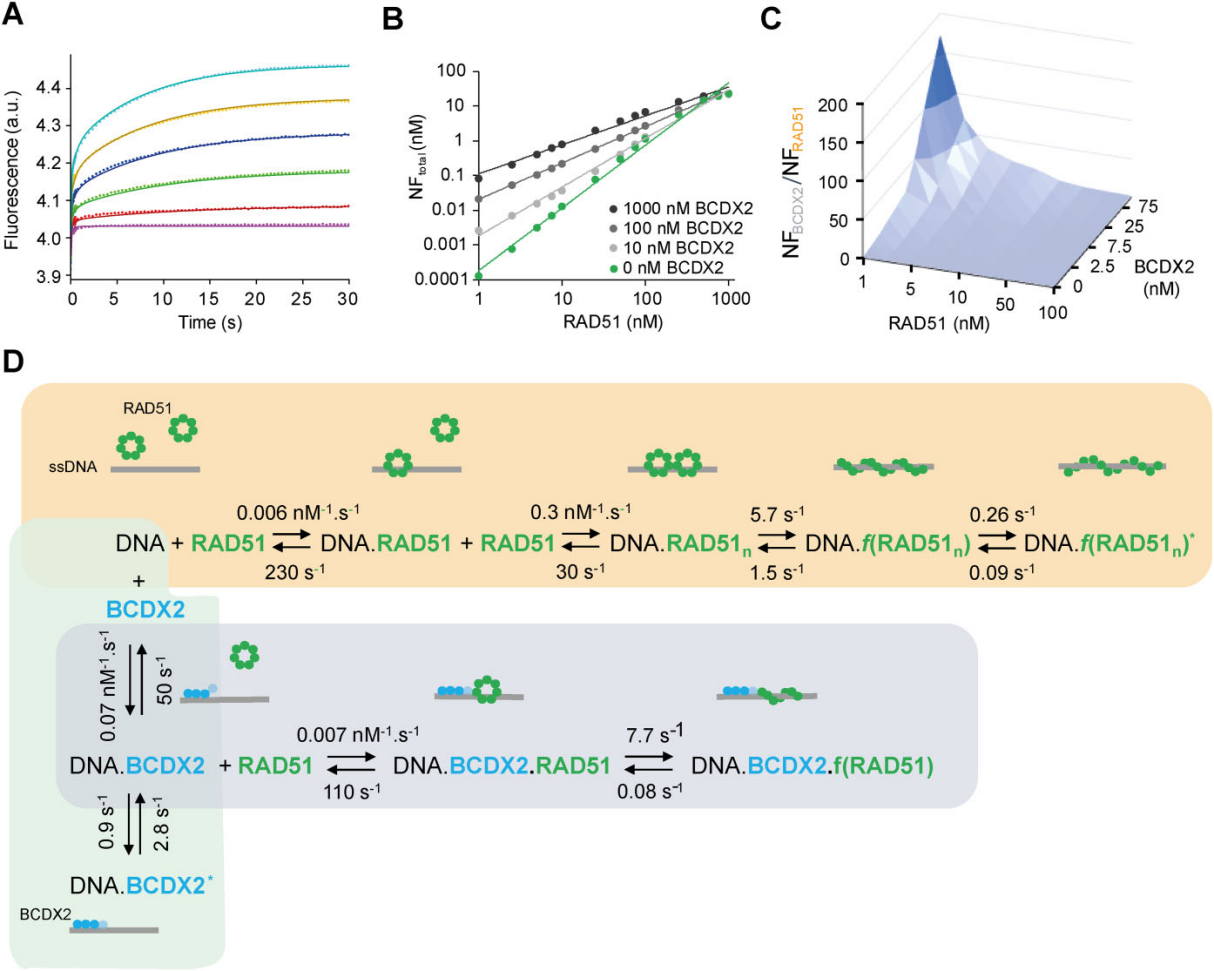
Mechanism of BCDX2-mediated loading of RAD51 on ssDNA. (**A**) Representative stopped-flow fluorescence data recorded upon the mixing of RAD51 (0–500 nM) with 30 nM Cy3-labeled DNA in the presence of 50 nM BCDX2. Global kinetic analysis of the cooperatively between BCDX2 and RAD51 included systematic data collection, with DNA titrated by RAD51 at 0, 25, 50 and 100 nM BCDX2, along with DNA titration by BCDX2 in the presence of 0, 200 and 300 nM RAD51 (complete kinetic analysis presented in Supplementary Figure S5). (**B**) The effect of BCDX2 concentration on the total equilibrium concentration of filament formation (NF_total_). Solid lines represent the fit to a power function, reflecting the exponential nature of the BCDX2 stimulatory effect at low concentration of RAD51. (**C**) The preferred route for nucleoprotein filaments formation (NF formed via BCDX2-mediated pathway (grey background) vs. RAD51 alone (orange background); NF_BCDX_/NF_RAD51_). Both (B) and (C) represent simulated data of RAD51 and BCDX2 interactions with 30 nM DNA obtained from the global numerical model (Supplementary Figure S8). (**D**) The kinetic model of simultaneous action of BCDX2 and RAD51. The rate constants for individual steps were determined by global numerical analysis. The complete set of experimental data included in the global kinetic analysis are summarized in Supplementary Figure S8. Given that oligomeric state of RAD51 during its loading on ssDNA remains unclear, we depicted it as a ring for simplicity. This representation, is meant to reflect possible polydisperse nature of RAD51, which may vary under different conditions.

In our pursuit to unravel the intricate mechanism underlying the ability of BCDX2 to promote RAD51 filament loading, we again used the potency of the stopped-flow assay. For this purpose, we collected a complex set of independent experimental data, including titration of DNA by RAD51 in both the absence and presence of various concentrations of BCDX, along with the titration by BCDX2 in the absence and presence of multiple RAD51 concentrations (Supplementary Figure S8B-K). The global kinetic analysis of the simultaneous action of BCDX2 and RAD51 (Figure 4D, green and orange) provided corresponding results when compared to previous independent analyses of individual interactions of BCDX2 (Figure 2C) or RAD51 (Figure 2F) with ssDNA. The overall performance and thermodynamic driving forces of individual pathways are visualized and compared in detail in the form of reaction coordinates (Supplementary Figure S9). Moreover, the complex global kinetic model (Figure 4D) allowed us to analyse the differences in the mechanism and kinetics of RAD51 interaction with DNA (orange background) compared to the combined action of both interacting molecules, RAD51 and BCDX2 (grey background). In the presence of BCDX2, the binding of RAD51 to DNA exhibited a single binding phase, starkly contrasting the stepwise cooperative binding of RAD51 to DNA in its absence. Importantly, while the rate of RAD51 binding in the presence of BCDX2 was not significantly faster than RAD51 binding alone, it displayed notably increased affinity (*K*_A_ for RAD51 binding to DNA was 0.026 nM^−1^ without BCDX2 and 0.064 nM^−1^ with BCDX2). Our kinetic model indicated a higher probability of RAD51 binding to the initial DNA.BCDX2 complex, as opposed to its interaction with the DNA.BCDX2* complex after isomerization. This suggests that the initial rapid binding of BCDX2 leads to the formation of the initial DNA.BCDX2 complex, which subsequently interacts with RAD51. In the absence of RAD51, the initial DNA.BCDX2 complex undergoes structural rearrangement leading to DNA.BCDX2*. Notably, in the presence of RAD51, the filament formation process is faster (5.7 s^−1^ without BCDX2 and 7.7 s^−1^ with BCDX2) and exhibits significantly higher filament stability (*K*_eq_ = *k*_on_/*k*_off_ = 7.7/0.08 = 96.3) compared to filament formation process with RAD51 alone (*K*_eq_ = 5.7/1.5 = 3.8)), which corresponds well to the results obtained using single molecule techniques. Moreover, our kinetic analysis did not detect any subsequent dynamical fluctuations of the RAD51 filament, highlighting further the stability of the nucleoprotein filament mediated by BCDX2. Intriguingly, our kinetic model shows that BCDX2’s loading effects were mostly pronounced at low RAD51 concentration (Figure 4B and C), in the concentration range where the previous analysis indicated that the cooperative mechanism of RAD51 alone is not effective (Figure 2E). Under these conditions, the BCDX2 loading pathway becomes the dominant route of filament formation (Figure 4D, grey background). At higher saturating concentrations of RAD51 (above 1 μM), the influence of BCDX2 diminished, and filament formation became independent of BCDX2 (Figure 4B and C).

### Loading by BCDX2 requires ATP-binding proficient RAD51 and stabilizes RAD51 on ssDNA

**Figure 5.**
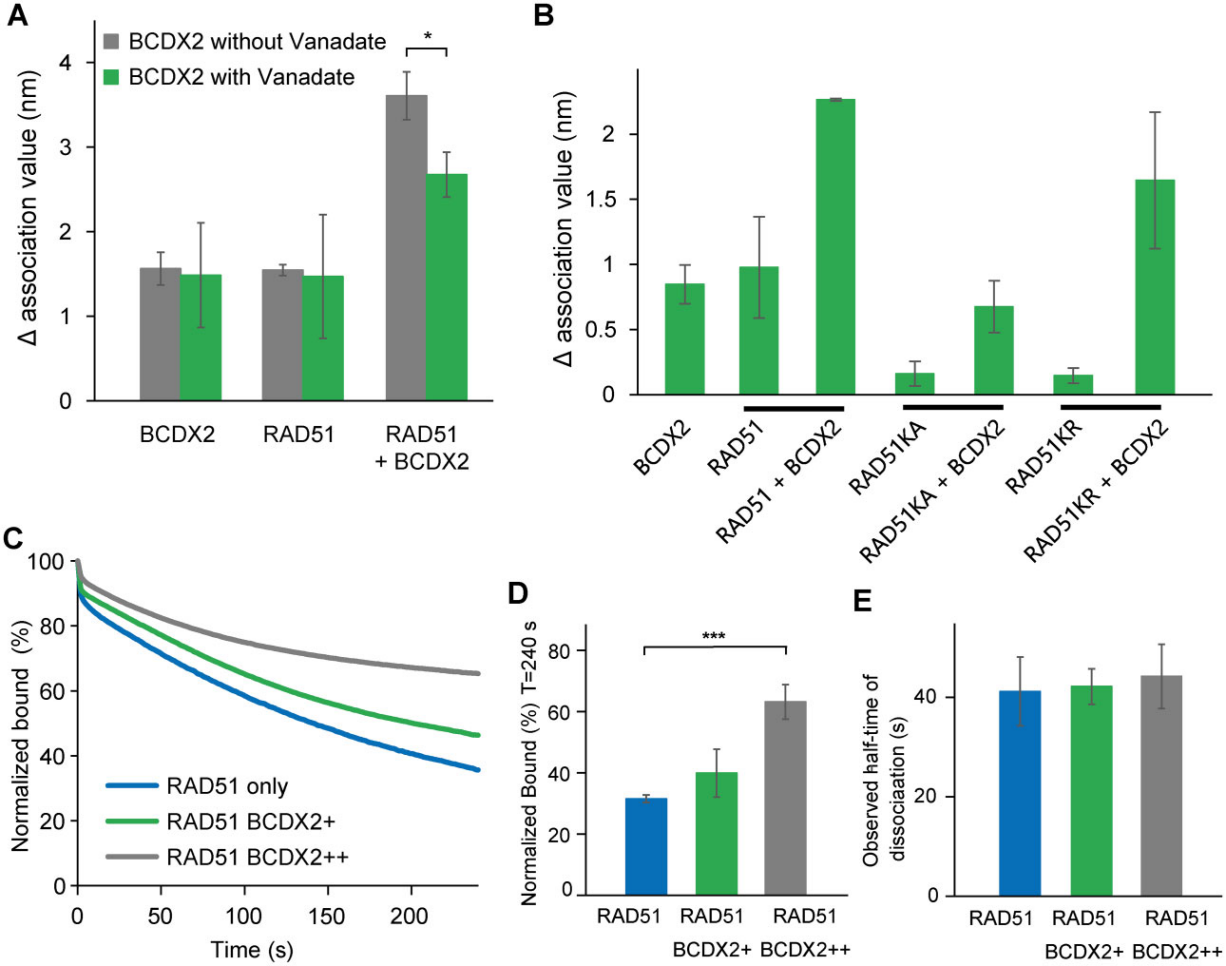
ATP hydrolysis of BCDX2 is required for RAD51 ssDNA loading. (**A**) Graph depicting changes in ssDNA (15 nM 5′-biotinylated ssDNA) binding amplitudes of RAD51 (600 nM) and BCDX2 (100 nM) and their mixture upon preincubation of BCDX2 with or without Vanadate to inhibit its ATPase activity. The values for association phase were normalized to the starting point (time point = 0 s). *n* = 3 independent experiments; data are means s.d. *P*-values are obtained by Student's *t*-test (two-tailed): * *P* ≤ 0.05. (**B**) Graph depicting changes in ssDNA (15 nM 5′-biotinylated ssDNA) binding amplitudes of BCDX2 (100 nM) and RAD51 (600 nM), including WT or Walker box mutants (RAD51 K133R (RAD51KR) or RAD51 K133A (RAD51KA)), and their mixtures. The values for association phase were normalized to the starting point (time point = 0 sec). data are presented as means s.d. (**C**) BLI sensorgrams obtained using RAD51-ssDNA filaments. The RAD51-ssDNA filaments preformed with RAD51 (600 nM) alone or in the presence of BCDX2 (50 or 100 nM) and 15 nM 5′-biotinylated ssDNA conjugated to streptavidin-loaded biosensors. The preformed protein-ssDNA filaments were then mixed with 100-fold excess unlabeled DNA and the dissociation was monitored. (**D**) Graph depicting changes in binding amplitudes of RAD51 and its mixture with two concentrations of BCDX2 during dissociation phase. The values for dissociation phase were normalized to the starting point (time point = 0 s). *n* = 3 independent experiments; data are means s.d. *P*-values are obtained by Student's *t*-test (two-tailed): *** *P* ≤ 0.001. (**E**) Graph of observed dissociation half-time of RAD51, BCDX2, or their mixture. *n* = 3 independent experiments; data are means s.d.

BCDX2 enhances RAD51 loading on ssDNA in the presence of ATP. To address the role of ATPase activity in RAD51 loading, we utilized BLI assay. Initially, we assessed the impact of Vanadate, a potent ATPase inhibitor, on RAD51 loading. Our results showed that the presence of Vanadate reduced RAD51 loading (Figure 5A), suggesting a requirement for ATP hydrolysis in this process. Next, we assessed the necessity of ATPase activity within RAD51 for its loading. To achieve this, we used two variants of RAD51: K131A and K131R mutants, deficient in ATP binding and hydrolysis, respectively. Interestingly, while we observed only a slight reduction in RAD51 loading in the presence of the K131R mutant, loading of the K131A mutant was completely abolished (Figure 5B). These data suggest that the loading of RAD51 by BCDX2 requires ATP-binding proficient RAD51 protein.

Furthermore, we also explored the ability of BCDX2 to stabilize RAD51 filament on ssDNA using BLI assay. Similar to the stabilization observed with RAD51 paralogs from *C. elegans* (23), we observed BCDX2-concentration dependent stabilization of RAD51 binding on ssDNA, with no change in the half-time of dissociation (Figure 5C–E). This indicates that while BCDX2 stabilizes RAD51 on ssDNA, it does not influence the mechanism of dissociation. These findings provide additional insight into the e mechanism of RAD51 loading and stabilisation by BCDX2.

### BCDX2 is proficient in loading RAD51 on short stretches of ssDNA

**Figure 6.**
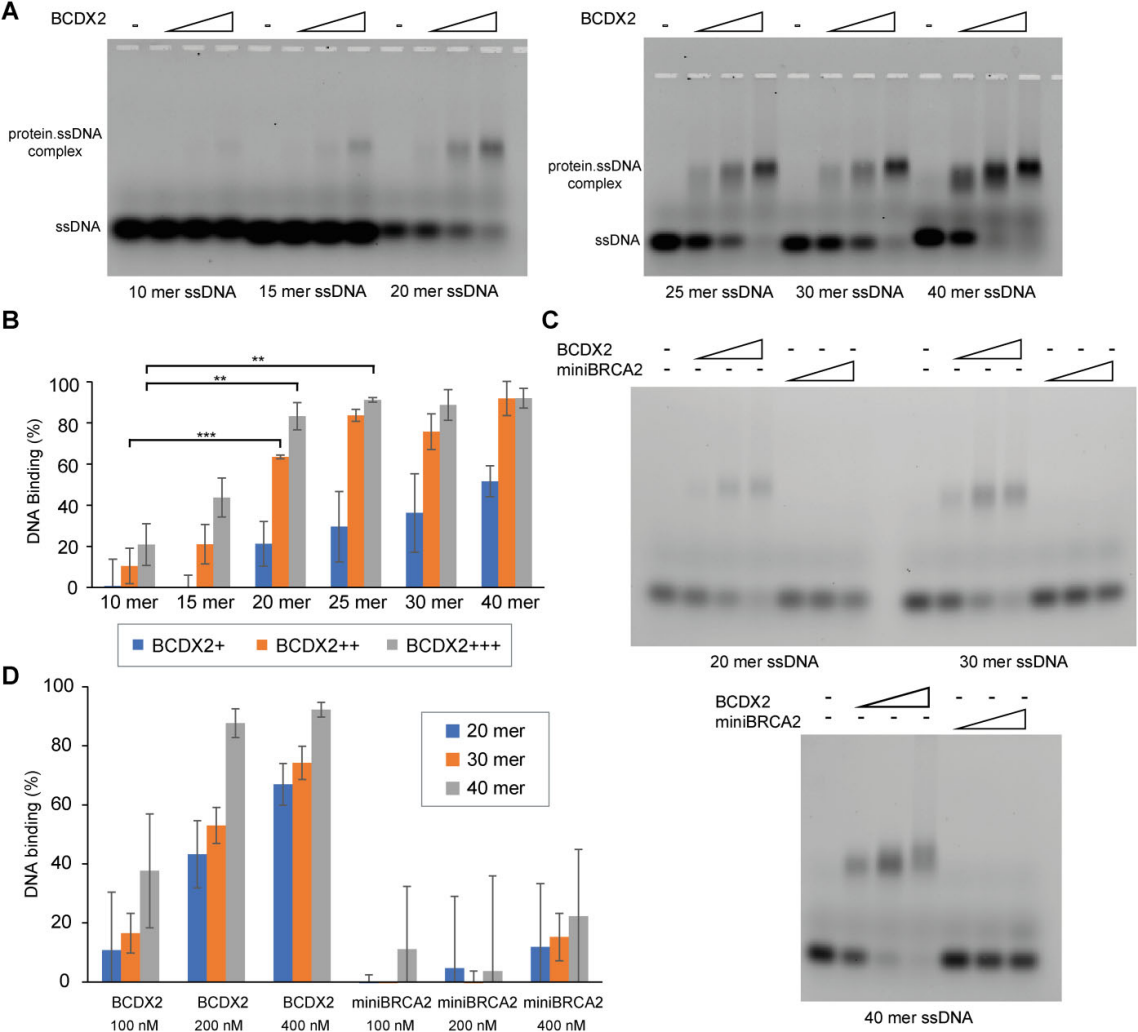
Comparison of BCDX2 and miniBRCA2 ssDNA binding. (**A**) Determination of the minimal ssDNA binding site size of BCDX2 using EMSA. Increasing concentrations of BCDX2 (100, 200 or 400 nM) were incubated with 20 nM ssDNA of various lengths (10–40 mer ssDNA). Protein-DNA complexes were crosslinked and analyzed on agarose gel. (**B**) Quantification of 100, 200 and 400 nM BCDX2 ssDNA binding by EMSA from (A). *n* = 3 independent experiments; data are presented as means s.d. *P*-values are obtained by Student's *t*-test (two-tailed): ** *P* ≤ 0.01, *** *P* ≤ 0.001. (**C**) Comparison of BCDX2 and miniBRCA2 binding to ssDNA of various lengths using EMSA. Increasing concentrations (100, 200 and 400 nM) of BCDX2 and miniBRCA2 were incubated with 20 nM ssDNA of various lengths (20, 30, and 40 nucleotides). Protein–DNA complexes were crosslinked and analyzed on agarose gel. (**D**) Quantification of ssDNA binding from (C). *n* = 3 independent experiments; data are presented as means s.d.

Given the well-documented role of BRCA2 in loading RAD51 on ssDNA (38–41), we sought to explore potential difference in the function of these RAD51 loaders. With the optimal RAD51 binding site identified as approximately three nucleotides per monomer, we anticipated that BCDX2 might have a preference for shorter ssDNA compared to BRCA2. To test this, we used EMSA with ssDNA of varying lengths (10–40 nucleotides) (Figure 6). Notable, BCDX2 demonstrated efficient binding to 20-mer, with significant binding observed even with 15-nucleotide-long ssDNA (Figure 6A and B). Furthermore, the length requirement can decrease even to 10nt when present within reversed replication fork (Supplementary Figure S10A and B), further supporting the difference in the binding modes for these substrates. To establish a comparison with BRCA2, we used miniBRCA2, a construct containing the ssDNA binding domain and known to partially complement BRCA2-deficient cells (42,34). In stark contrast to miniBRCA2, BCDX2 showed stronger binding to all tested substrates using either EMSA or BLI assays (Figure 6C and D, and Supplementary Figure S10C–E), underscoring profound affinity of BCDX2 for short ssDNA.

**Figure 7.**
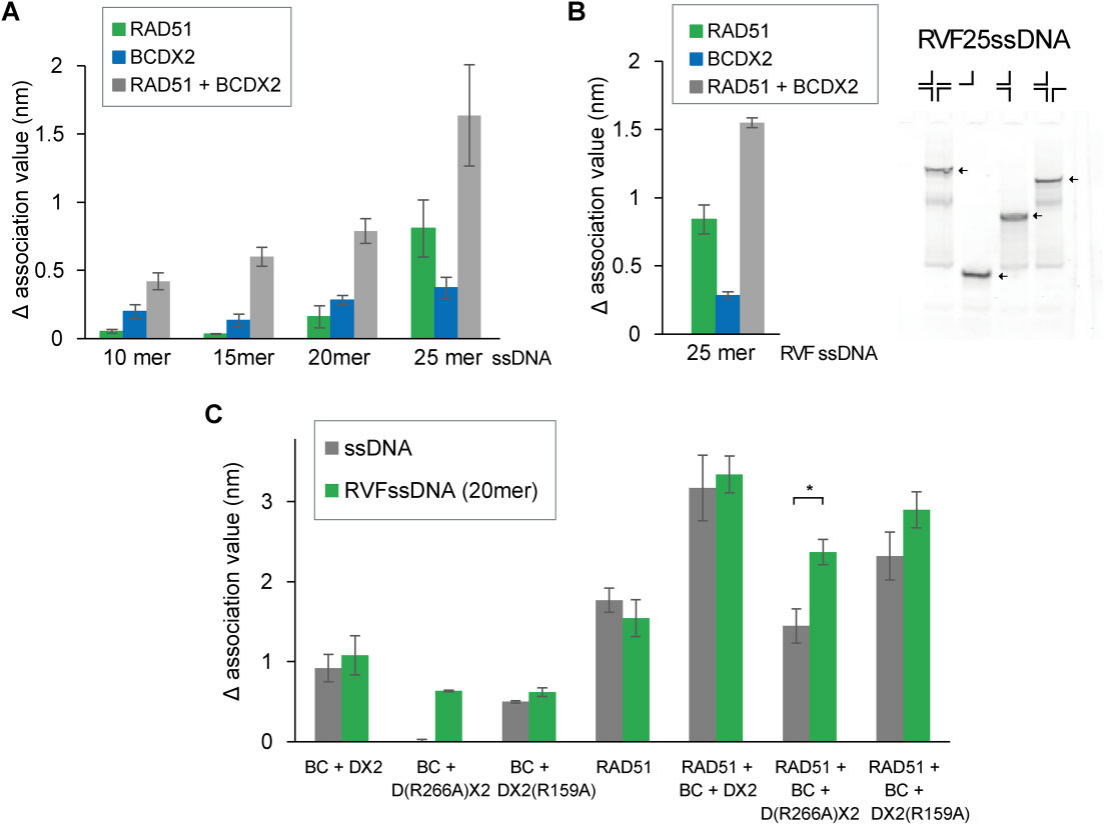
BCDX2-mediated loading of RAD51 on short ssDNA and reversed replication fork. (**A**) Graph depicting changes in binding amplitudes of 600 nM RAD51, 100 nM BCDX2 or their mixture on ssDNA of various lengths (10, 15, 20 and 25 nucleotides). A solution of RAD51, BCDX2 or their mixture was loaded onto streptavidin-loaded biosensors conjugated with 15 nM corresponding 5′-biotinylated ssDNA, and association was monitored. The values for the association phase were normalized to the starting point (time point = 0 s). *n* = 3 independent experiments; data presented as means s.d. (**B**) Graph depicting changes in binding amplitudes of RAD51, BCDX2, and their mixture on RVF with 25 nt long ssDNA. *n* = 3 independent experiments; data presented as means s.d. Gel representing the formation of Cy3-labeled reversed fork DNA with 25nt ssDNA, ssDNA, Y-fork, and partially reversed fork DNA are shown too as controls. The DNA substrates in the gel are indicated by arrowheads. (**C**) Graph depicting changes in binding amplitudes of 600 nM RAD51, 100 nM BCDX2 containing WT or mutant variants of DX2 (D(R266A)X2 and DX2(R159A)), or their mixture on 20 mer ssDNA or RVF with 20 mer ssDNA. A solution of RAD51, three BCDX2 variants, or their mixture was loaded onto streptavidin-loaded biosensors conjugated with 15 nM corresponding 5′-biotinylated DNA, and association was monitored. The values for the association phase were normalized to the starting point (time point = 0 s). *n* = 3 independent experiments; data presented as means s.d.

To verify that BCDX2 is capable of loading RAD51 onto short DNA, we used again BLI assay with 10, 15, 20 and 25-mer biotinylated-DNA. Employing corresponding non-saturating concentrations of RAD51, BCDX2 or their combination, we monitored the dynamics of protein association in the presence of ATP and Mg^2+^ (Figure 7A). Examination of the normalized association phases revealed an increase in amplitude of RAD51 association in the presence of BCDX2, suggesting that BCDX2 facilitates the binding of RAD51 to ssDNA stretches as short as 15 nucleotides. In summary, BCDX2 shows a higher affinity for short ssDNA compared to miniBRCA2 and demonstrates the ability to load RAD51 onto such a short DNA stretches, suggesting a potential functional distinction in the action of these RAD51 loader protein.

### BCDX2 promotes RAD51 loading on reversed replication fork

To further explore potential difference between BRCA2 and BCDX2, we next asked whether ability of BCDX2 to bind substrates mimicking reversed replication forks could also facilitate loading RAD51 on such substrate. Indeed, using BLI assay, we observed enhanced loading of RAD51 on reversed replication forks with 25mer-ssDNA tail (Figure 7B). This data indicates that the binding of BCDX2 to reversed fork substrate can also promote RAD51 loading.

Subsequently, we examined the D(R266A)X2 and DX2(R159A) mutants, which, despite being defective in binding ssDNA, still demonstrated binding to reversed replication fork. Notably, using BLI assay, these mutants did not show any RAD51 loading onto ssDNA (Figure 7C). However, they retained the capacity to load RAD51 on substrate mimicking reversed replication fork (Figure 7C), indicating a functional divergence between binding to ssDNA and branch DNA substrates and providing additional specificity for loading of RAD51.

## Discussion

The formation and precise regulation of RAD51 and its nucleoprotein filament on ssDNA represent a key step not only in repair of DSBs, but also during the intricate orchestration of stalled replication forks. In the later context, RAD51’s multifaceted function encompasses safeguarding nascent and parental DNA from degradation, promoting replication fork regression, and aiding in fork restart. Failures to properly assemble or regulate RAD51 represent serious implications for genome stability problems and have been associated with the development of various cancers and Fanconi aneamia (43). Here, we focus on the mechanistic characterization of RAD51 paralogs, specifically the BCDX2 complex, in intricate regulation of RAD51 protein.

**Figure 8.**
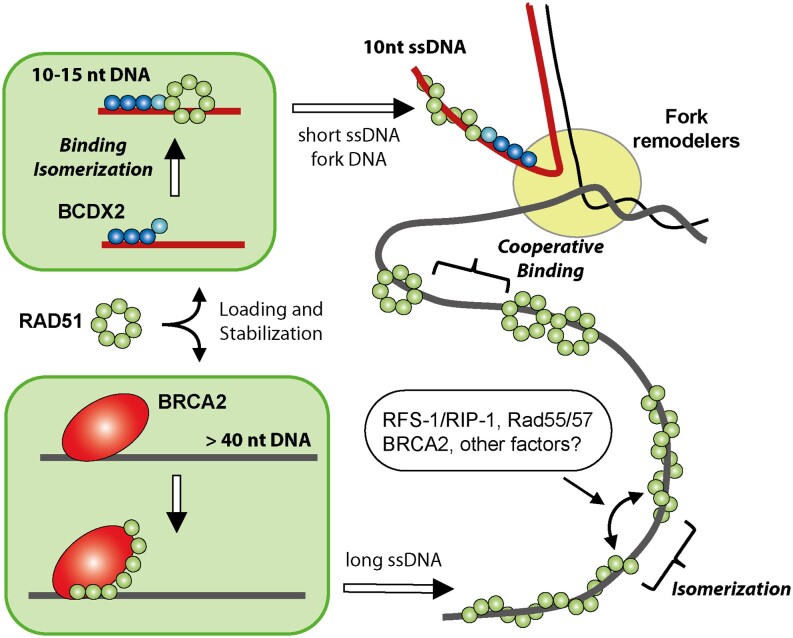
BCDX2 complex promotes loading and stabilization of RAD51 filament on short ssDNA and reversed replication fork. RAD51 shows cooperative binding to DNA, characterized by two binding and two isomerization steps. The equilibrium between the two forms of RAD51 filament may be influenced by other factors (i.e. BRCA2, *C. elegans* RFS-1/RIP-1, *S. cerevisiae* Rad55/57). In contrast, BCDX2 binding to ssDNA is represented by a single binding step followed by a conformational change, suggesting a possible role for post-translational modification (PTM) in regulation of this process. In the presence of BCDX2, the initial rapid binding of BCDX2 to ssDNA actively promotes RAD51 loading onto ssDNA, characterized by a single binding and isomerization step. The ability of BCDX2 to bind short ssDNA and fork-like substrates, in contrast to BRCA2, enables the loading of RAD51 onto short ssDNA stretches, facilitating replication fork reversal and DSB repair. Given that oligomeric state of RAD51 during its loading on ssDNA remains unclear, we depicted it as a ring for simplicity. This representation, is meant to reflect possible polydisperse nature of RAD51, which may vary under different conditions.

Recent structural studies have shed light on the structure and function of BCDX2 paralog complex (31,32). Our findings unveil that the BCDX2 complex operates through an induced-fit ssDNA binding mechanism, a marked contrast to the cooperative binding mechanism exhibited by RAD51 (Figure 8). This differential binding behaviour likely contributes to the distinct roles of these complexes in HR. Using a range of biochemical and biophysical approaches, we characterized the functional impact of the BCDX2 complex on RAD51 filament formation. We demonstrate that the BCDX2 complex suppresses the cooperativity requirement by facilitating the loading of RAD51 on ssDNA and accelerating filament growth and stability. This nicely aligns with our negative staining and recently reported cryo-EM analysis of the human BCDX2 complex revealing a linear arrangement of subunits, distinct from the RAD51 ring-like structure and with RAD51C/RAD51D/XRCC2 mimicking the behaviour of three RAD51 protomers within a nucleoprotein filament (31,32). We also provide supporting evidence that the loading of RAD51 requires ATPase activity of BCDX2 complex (31) and show that it depends on ATP-binding status of RAD51 protein. Notably, in the absence of BCDX2, RAD51 filament formation results in establishment of an equilibrium between two filament forms, a transition characterized by isomerization. These forms may correspond to the previously observed extended and compressed RAD51 filaments (44). Intriguingly, in the presence of BCDX2, only one major filament type is observed, suggesting a preferred structural arrangement and the potential for regulation of these transitions by various RAD51 interaction partners (Figure 8). While our study did not provide evidence for the previously suggested incorporation of RAD51 paralogs within the filament (27), we cannot rule out this possibility considering the limitations imposed by the size of ssDNA used in this study. Additionally, there is ongoing debate regarding the oligomeric state of RAD51 and its relevance to filament formation, which warrants further investigation to clarify this point (45–47).

Previously, significant emphasis was placed on the role of BRCA2 in loading RAD51 and promoting filament formation (39,48). However, our research highlights a crucial distinction - the BCDX2 complex possesses a smaller DNA binding size, corresponding to 15–20 nucleotides, and exhibits a higher affinity for smaller ssDNA compared to miniBRCA2 (Figure 8). This characteristic aligns with BCDX2’s ability to load RAD51 onto short stretches of DNA and provides a plausible explanation for its selective requirement in fork reversal, which is BRCA2-independent (28,49–51). Fork reversal is expected to involve creation of short nascent ssDNA and we propose that BCDX2 likely plays a crucial role in promoting RAD51 filament formation during this process. Indeed, we show that BCDX2 can load RAD51 on reversed replication fork substrate, which stems from BCDX2 affinity for branched DNA structures, with significant contributions from RAD51D and XRCC2 subunits. Moreover, while mutants defective in ssDNA binding retain the ability to bind branched DNA substrates, they still facilitate RAD51 loading onto reversed replication forks. This observation indicates potentially two DNA binding modes with fork substrates providing additional stabilization as apparent also from observed conformational change by EM upon fork binding. Role of BCDX2-mediated loading of RAD51 in context of reversed forks gains further support from the direct interaction of BCDX2 and specialised enzymes like SMARCAL1 and ZRANB3, which provide motor-drive activity during fork reversal (37,51–54) (Figure 8). In contrast, BRCA2 may require longer ssDNA not only for efficient RAD51 filament loading but also its stabilization (13,28,55), ensuring the protection of stalled/reversed forks (51,56). Similarly, BCDX2 complex can also contribute to filament stabilization *in vitro* and *in vivo*, as reported for *C. elegans* Rfs-1/Rip-1 and *S. cerevisiae* Rad55/Rad57 complexes (23,27). Given the role of BCDX2 also in repair of DSBs (13,15,16), where the average length of ssDNA generated during resection is typically in kb of ssDNA (57,58), we suggest that BCDX2 may still be required for RAD51 loading in situations involving limited resection due to structural or topological constrains.

In conclusion, our study provides first mechanistic description for the critical role of BCDX2 in the initial step of RAD51 filament assembly, highlighting its function in promoting HR-mediated DNA repair and processing of stalled replication forks. This work unveiled a novel layer of complexity in the regulation of RAD51’s multifaceted role in HR and genome maintenance. Beyond advancing our fundamental understanding of DNA repair mechanisms, comprehending the molecular roles of these complexes holds significant implications for cancer susceptibility and therapeutic intervention.

## Supplementary Material

gkae770_Supplemental_File

## Data Availability

The data within this article are available in the article, its online Supplementary material, or will be shared upon reasonable request to the corresponding author.
