# Supplementary material for: Mechanism of BCDX2-mediated RAD51 nucleation on short ssDNA stretches and fork DNA
Source: Nucleic Acids Res. 2024 Sep 12;52(19):11738–52. doi: 10.1093/nar/gkae770 (PMC11514458; doi:10.1093/nar/gkae770)

**Supplemental material**

**Mechanism of BCDX2-mediated RAD51 nucleation on short ssDNA stretches and fork DNA**

Masaki Akita, Paul Girvan, Mario Spirek, Jiri Novacek, David Rueda, Zbynek Prokop, and Lumir Krejci

**Supplemental Experimental Procedures**

**Kinetic Data Analysis and Statistics.**

The kinetic data were fit globally with the KinTek Explorer program (KinTek, USA). This dynamic kinetic simulation program allowed multiple data sets to be ﬁt simultaneously to a single model. Data fitting used numerical integration of rate equations from an input model (Figure 2, Supplementary Figures S5, S6 and S7) searching a set of parameters applying the Bulirsch–Stoer algorithm with an adaptive step size that produces a minimum χ^2^ value calculated by using nonlinear regression based on the Levenberg-Marquardt method ^1^. Residuals were normalized by sigma value for each data point. The standard error of the fitted parameters was calculated from the covariance matrix during nonlinear regression. The observable signal (*FI*) was defined as the sum of the contributions of all involved DNA species to the total Cy3 fluorescence. The Equation 1 was used to define the signal collected upon mixing BCDX2 with DNA, where *f* scales the signal to concentration of DNA and sensitivity of the measurement, factor *a* defines the relative change in fluorescence in forming DNA.BCDX2 and DNA.BCDX2^*^ complexes. The Equation 2 was used to define the signal collected upon mixing RAD51 with DNA, where *f* scales the signal to concentration of DNA and sensitivity of the measurement, factor *a* defines the relative change in fluorescence upon RAD51 binding and forming DNA.RAD51_n_ bound complexes, factors *b* and *c* define the relative change in fluorescence in forming filament DNA.*f*(RAD51_n_) and its conformational rearrangement to DNA.*f*(RAD51_n_)^*^, respectively. The Equation 3 was used to define the signal of combined experiments mixing DNA with both BCDX2 and RAD51.

$${FI}_{BCDX2} =f\times(DNA+a\times\left( DNA.BCDX2+{DNA.BCDX2}^{*} \right))$$

*(Eq. 1)*

$${FI}_{RAD51} =f\times\left( DNA+a\times\left( DNA.RAD51+DNA.{RAD51}_{n} \right)+b\times DNA.{f(RAD51}_{n} \right)+ c\times{DNA.{f(RAD51}_{n})}^{*})$$

*(Eq. 2)*

$${FI}_{BCDX,RAD51} =f\times\left( DNA+a\times\left( DNA.RAD51+DNA.{RAD}_{51n}+ DNA.BCDX2.RAD51 \right)+b\times(DNA.{f(RAD51}_{n} \right)+DNA.BCDX2.f(RAD51))+ c\times{DNA.f(RAD51)}^{*})$$

*(Eq. 3)*

The free energy profiles were constructed based upon the kinetic data using the Eyring equation (Equation 5) at reference temperature *T* = 310 K, and reference concentration of 2 µM RAD51 and 400 nM BCDX2. *R* is the universal gas constant, *k*_B_ is the Boltzmann constant, h is Planck's constant, and *k* is the rate.

$$\Delta G^{\ddagger}=-R.T.ln(k.h/k_{B}.T))$$

*(Eq. 5)*

##

## **Conventional Analytical Fitting of Kinetic Data**

We initiated the kinetic analysis by employing conventional analytical curve fitting on stopped-flow data. This approach aimed to discern the primary characteristics of potential kinetic models and derive preliminary estimates for specific rate and equilibrium constants (**Supplementary Figure S7**). Upon mixing BCDX2 with DNA (**Supplementary Figure S7A left**), the fluorescence intensity was increased, exhibiting two kinetic phases that fit a double exponential function (**Equation 1**).

$$f=F_{0}+A_{1}.\left( 1-e^{-k_{fast}.t} \right)+A_{2}.(1-e^{-k_{slow}.t})$$

(Eq. 1)

The rate of the fast phase (*k*_fast_) exhibited a pronounced dependence on the concentration of BCDX2 (**Supplementary Figure S7A middle**), indicating that this is an associative step involving BCDX2 binding to DNA. The rate of the second slow phase (*k*_slow_) shows no significant dependence on BCDX2 concentration, indicating that this second step is a unimolecular isomerization that follows the initial binding (**Scheme 1**). Fitting the concentration dependence of *k*_fast_ using linear rate equation (**Equation 2**) provided initial estimates of the rate constants for the association (*k*_1_ = 0.077 ± 0.006 nM^-1^ s^-1^) and an approximate value for the rate constant of dissociation (*k*_-1_ ~ 36 s^-1^) of the initial DNA-BCDX2 complex.

$$DNA+BCDX2\underset{\leftrightarrow}{k_{1};k_{-1}}DNA.BCDX2\underset{\leftrightarrow}{k_{2};k_{-2}}{DNA.BCDX2}^{*}$$

(Scheme 1)

$$k_{fast}=\left[ BCDX2 \right].k_{1}+k_{-1}+k_{2}+k_{-2}$$

(Eq. 2)

The second slow phase (*k*_slow_) provides a rough estimate for the velocity of the following isomerization step ~ 6 s^-1^ which is a combination of the effect of both forward and reverse rates.

The concentration dependence of the amplitudes (**Supplementary Figure S7A right**) exhibits a purely hyperbolic relationship, further indicating simple or binding kinetics. Fitting the hyperbola (**Equation 3**) to the concentration dependence of the amplitude of the first phase (*A*_1_) made it possible to obtain an initial estimate of the dissociation constant for the initial DNA.BCDX2 complex *K*_d,1_ = *k*_-1_/*k*_1_ = 310 ± 50 nM. Fitting the concentration dependence of the total amplitude (*A*_TOT_ = *A*_1_ + *A*_2_) provided an estimate of the overall dissociation constant for the two-step binding *K*_d,net_ = 220 ± 20 nM. The observed decrease in the overall dissociation constant, denoted as *K*_d,net_, is a characteristic phenomenon in two-step reactions when an induced unimolecular step occurs after the initial binding, thereby kinetically enhancing the stability of the bound complex and subsequently increasing its concentration compared to free reaction partners (**Equation 4**).

$$A_{i}= \frac{A_{lim}.\left[ BCDX2 \right]}{\left[ BCDX2 \right]+ K_{d,i}}$$

(Eq. 3)

$$K_{d,net}= \frac{k_{-1}.k_{-2}}{k_{1}.(k_{2}+k_{-2})}$$

(Eq. 4)

The conventional analytical fitting was also performed for DNA and RAD51 binding (**Supplementary Figure S7B**). The kinetics of DNA binding to RAD51 show a more complex character. Fluorescence data obtained using stopped-flow show at least three distinct phases (**Supplementary Figure S7B left**) that fit a triple exponential function (**Equation 5**).

$$f=F_{0}+A_{1}.\left( 1-e^{-k_{obs1}.t} \right)+A_{2}.\left( 1-e^{-k_{obs2}.t} \right)+A_{3}.(1-e^{-k_{obs3}.t})$$

(Eq. 5)

The rate of the initial phase of the interaction between DNA and RAD51 (*k*_obs1_) exhibits a pronounced dependence on concentration and thus can be unambiguously assigned as an association event. In contrast to BCDX2, the concentration dependence of the binding kinetics in RAD51 exhibits complex behavior (**Supplementary Figure S7B middle**). The quadratic trend indicates that within this step, the binding process involves more than one molecule of RAD51, and subsequently, the binding steps manifest a cooperative characteristic. To determine the extent of cooperativity, the concentration dependence of the initial phase amplitude (*A*_1_) was fit to the Hill model (**Equation 6**) and the amplitude of the second (*A*_2_) and the third (*A*_3_) phase to extended Hill model (**Equation 7**) to account the impact of the preceding step on the signal (**Supplementary Figure S7B right**). Note that all three steps compete for the same signal source. Fitting the equilibrium amplitude calculated from the first, second and third phases observed in the fluorescence data yielded Hill coefficient n_1_ = 2.0 ± 0.1, n_2_ = 2.1 ± 0.4 and n_3_ = 2.3 ± 0.4, respectively. The value n indicates the number of cooperative binding events (n = 2).

$$A_{1}= \frac{A_{1,lim}.\left[ RAD51 \right]^{n}}{\left[ RAD51 \right]^{n}+ {K_{d,1}}^{n}}$$

(Eq. 6)

$$A_{i}= \frac{A_{i,lim}.\left[ RAD51 \right]^{n}}{\left[ RAD51 \right]^{n}+ {K_{d,i}}^{n}}+\frac{A_{i-1, lim}.\left[ RAD51 \right]^{n}}{\left[ RAD51 \right]^{n}+ {K_{d,i-1}}^{n}}$$

(Eq. 7)

$$k_{obs}= k_{off}+k_{on}.\left[ RAD51 \right]^{n}$$

(Eq. 8)

These results thus lead to the definition of a minimal kinetic mechanism, containing four steps, which elucidates the experimental observations obtained during the analysis of DNA and RAD51 binding (**Scheme 2**). For clarity, it is essential to emphasize that the first association phase, out of the three exponential phases observed in the fluorescence data, encompasses at least two individual steps explaining the cooperative manner of RAD51 loading onto the DNA.

$$DNA+n.RAD51\underset{\leftrightarrow}{k_{1};k_{-1}}\ldots\underset{\leftrightarrow}{k_{n};k_{-n}}{DNA.(RAD51)}_{n}\underset{\leftrightarrow}{k_{3};k_{-3}}{DNA.f(RAD51}_{n})\underset{\leftrightarrow}{k_{4};k_{-4}}{{DNA.f(RAD51}_{n})}^{*}$$

(Scheme 2)

Fitting the concentration dependence of *k*_obs1_ to **Equation 8** provided an estimate of the apparent rate of the DNA.RAD51 complex dissociation *k*_off_ = 38 ± 2 s^-1^. The inaccuracy of the analytical fitting and the propagation of the errors in the power manner (function of [conc.]^n^) did not allow for a more precise determination of the value of the apparent rate of association (*k*_on_). The concentration dependence of the observed rates of both subsequent fluorescence phases does not exhibit significant concentration dependence, indicating two unimolecular processes involving slow conformational changes in the bound complex (isomerization processes). The data acquired from the second (*k*_obs2_) and third (*k*_obs3_) fluorescence phases yielded initial estimates 2.5 and 0.16 s⁻¹, respectively, for the velocities of the isomerization steps that succeed RAD51 binding.

Further examination of the concentration dependence of the amplitude provided additional support for the proposed four-step mechanism, indicating interrelated processes underlying all observed phases in the fluorescence kinetic data. The application of the Hill model yielded progressively decreasing values for the individual dissociation constants: *K*_d,1_ = 1.6 ± 0.2 μM, *K*_d,2_ = 1.0 ± 0.3 μM and *K*_d,3_ = 0.7 ± 0.1 μM. These findings suggest a gradual enhancement in the stability of the DNA.RAD51 complex, attributed to successive isomerization processes.

Although the analytical fitting provides valuable information on the mechanism, particularly through the analysis of the concentration dependence of the rates and amplitudes, it is limited in its ability to provide precise parameter estimates due to approximations and error accumulation during multistep fitting ^2^. To overcome these limitations, we modelled the kinetic data globally using numerical integration of the rate equations derived from the proposed kinetic models. The parameters from the analytical fit were used as starting values for the global fit.

**Single-molecule FRET (smFRET) acquisition and analysis**

The DNA construct was prepared by annealing 1 µM each of the 3′-biotin 5′-Cy3 anchor and the Cy5 poly-dT7 DNA (Integrated DNA Technologies) (Supplementary Table 1) in annealing buffer (50 mM Tris-HCl, pH 7.5, 50 mM NaCl, 5 mM MgCl_2_) at 95˚C for 60 s and cooling to room temperature over 30 min.

Single-molecule FRET experiments were performed as described ^3,4^. Briefly, quartz microscope slides (UQC optics) and glass coverslips were passivated with methoxy-PEG-SVA (Mr = 5,000; Laysan Bio Inc.) doped with 5% biotin-PEG-SVA (Mr = 5,000; Laysan Bio Inc.) to minimize non-specific binding to the surface.

Flow chambers were prepared by sandwiching a quartz slide and glass coverslip together using 0.12 mm thick double-sided adhesive sheets (Grace-Bio-Labs SecureSeal). Neutravidin (0.1 mg/ml) in T50 buffer (50 mM Tris-HCl pH 7.5, 50 mM NaCl) was injected into the assembled flow chamber and incubated for 5 min to allow binding to the biotinylated PEG surface. Excess neutravidin was washed out with experiment buffer (50 mM Tris-HCl, pH 7.5, 50 mM NaCl, 5 mM MgCl_2_, 1mM CaCl_2_).

The DNA construct was surface immobilized by diluting to 5 pM in experiment buffer, injecting into the flow chamber and incubating for 5min. Excess DNA was washed out with imaging buffer (50 mM Tris-HCl, pH 7.5, 50 mM NaCl, 5 mM MgCl_2_, 1mM CaCl_2_, 2 mM ATP, 0.2 mg/mL BSA, 2 mM Trolox to prevent photoblinking of the dyes, and an oxygen scavenger system consisting of 2.5 mM 3,4-dihydroxybenzoic acid (PCA) (Sigma) and 250 nM protocatechuate dioxygenase (PCD) (Sigma) to minimize photobleaching). Finally, the proteins for each experiment (100 nM RAD51 and 25 nM BCDX2) were introduced in imaging buffer.

Fluorescence trajectories from single molecules were manually inspected using custom MATLAB code. Molecules that displayed single step photobleaching of the acceptor and/or donor were included in further analysis, carried out in tMAVEN ^5^. Control experiments with only DNA present displayed a single high FRET state without transitions to lower FRET states. For experiments where BCDX2 and/or RAD51 was present, any trajectory that displayed a single high FRET state without transitions, (i.e., a trace consistent with DNA alone) was rejected, and only trajectories that displayed transitions or lower FRET were included. Trajectories were truncated to the region prior to photobleaching and analysed with a Hidden Markov model (HMM) using vbConsensus with model selection. A four-state model best described the data. FRET histograms with the HMM model shown were plotted in tMAVEN. The rates of transition between each of the states identified were determined directly from the HMM transition matrix. The overall rate for filament growth was calculated as the mean time needed to traverse through each off the four FRET states, from high FRET to low FRET. The stepwise transition from high FRET (state 1) to low FRET (state 4) can be described as,

$1 \begin{matrix} k_{1} \\ \rightleftharpoons\\ k_{-1} \end{matrix} 2 \begin{matrix} k_{2} \\ \rightleftharpoons\\ k_{-2} \end{matrix} 3 \begin{matrix} k_{3} \\ \rightleftharpoons\\ k_{-3} \end{matrix}$4

with individual rate constants $k_{i}$. The mean time needed for the forward transition $\left\langle t_{p} \right\rangle$ (i.e., filament growth) can be calculated as described in^6^ and is given by

$$\left\langle t_{p} \right\rangle=\frac{1}{k_{1}}\left[ 1+\frac{k_{-1}}{k_{2}}+\frac{k_{-1}k_{-2}}{k_{2}k_{3}} \right]+\frac{1}{k_{2}}\left[ 1+\frac{k_{-2}}{k_{3}} \right]+\frac{1}{k_{3}}$$

| DNA Substrates | | 5' labeled | Int labeled | 3' labeled | Length | Sequence |
| --- | --- | --- | --- | --- | --- | --- |
| EM-analysis | |  |  |  | 49 mer | AGCTACCATGCCTGCACGAATTAAGCAATTCGTAATCATGGTCATAGCT |
|  | ssDNA |  |  |  |  |  |
|  | Reversed fork ssDNA (RVF1) | | |  |  |  |
|  |  |  |  |  | 50 mer | TGCCGAATTCTACCAGTGCCAGTGATGGACATCTTTGCCCACGTTGACCC |
|  |  |  |  |  | 50 mer | TGGGTCAACGTGGGCAAAGATGTCCTAGCAATGTAATCGTCTATGACGTT |
|  |  |  | FITC labeled  Z position | | 49 mer | TCGGATCCTCTAGACAGCTCZCATGATCACTGGCACTGGTAGAATTCGGC |
|  |  |  |  |  | 25 mer | CAACGTCATAGACGATTACATTGCT |
| EMSA |  |  |  |  |  |  |
|  | ssDNA |  |  |  |  |  |
|  |  |  |  | FITC | 49 mer | AGCTACCATGCCTGCACGAATTAAGCAATTCGTAATCATGGTCATAGCT |
|  |  | FITC |  |  | 10 mer | AGAAGAGAGC |
|  |  | FITC |  |  | 15 mer | AGAAGAGAGCAGAGG |
|  |  | FITC |  |  | 20 mer | GAATGTGTGTCTCAATC |
|  |  | FITC |  |  | 25 mer | AGCTATGACCATGATTACGAATTGC |
|  |  | FITC |  |  | 27 mer | AGCTATGACCATGATTACGAATTGCTT |
|  |  | FITC |  |  | 30 mer | GTACAAAGCTCTGGCATGATACTATGCGGC |
|  |  | FITC |  |  | 40 mer | CTAAGTTCGTCAGGATTCCAGCATTCTAACAGTCATAGCG |
|  | dsDNA |  |  |  |  |  |
|  |  |  |  | FITC | 49 mer | AGCTACCATGCCTGCACGAATTAAGCAATTCGTAATCATGGTCATAGCT |
|  |  |  |  |  | 49 mer | AGCTACCATGCCTGCACGAATTAAGCAATTCGTAATCATGGTCATAGCT |
|  | Reversed fork ssDNA (RVF1) | | |  |  |  |
|  |  |  |  |  | 50 mer | TGCCGAATTCTACCAGTGCCAGTGATGGACATCTTTGCCCACGTTGACCC |
|  |  |  |  |  | 50 mer | TGGGTCAACGTGGGCAAAGATGTCCTAGCAATGTAATCGTCTATGACGTT |
|  |  |  | FITC labeled  Z position | | 49 mer | TCGGATCCTCTAGACAGCTCZCATGATCACTGGCACTGGTAGAATTCGGC |
|  |  |  |  |  | 25 mer | CAACGTCATAGACGATTACATTGCT |
|  | Reversed fork dsDNA (RVF2) | | |  |  |  |
|  |  |  |  |  | 50 mer | TGCCGAATTCTACCAGTGCCAGTGATGGACATCTTTGCCCACGTTGACCC |
|  |  |  |  |  | 50 mer | TGGGTCAACGTGGGCAAAGATGTCCTAGCAATGTAATCGTCTATGACGTT |
|  |  |  |  |  | 49 mer | TCGGATCCTCTAGACAGCTCCATGATCACTGGCACTGGTAGAATTCGGC |
|  |  | FITC |  |  | 50 mer | CAACGTCATAGACGATTACATTGCTTCATGGAGCTGTCTAGAGGATCCGA |
|  | Y-form |  |  |  |  |  |
|  |  |  |  | FITC | 49 mer | AGCTACCATGCCTGCACGAATTAAGCAATTCGTAATCATGGTCATAGCT |
|  |  |  |  |  | 47 mer | AGCTATGACCATGATTACGAATTGCTTGGAATCCTGACGAACTGTAG |
|  | GAP |  |  |  |  |  |
|  |  |  |  |  | 90 mer | AAATCAATCTAAAGTATATATGAGTAAACTTGGTCTGACAGTTACCAATGC  TTAATCAGTGAGGCACCTATCTCAGCGATCTGTCTATTT |
|  |  | FITC |  |  | 45 mer | AAATAGACAGATCGCTGAGATAGGTGCCTCACTGATTAAGCATTG |
|  |  |  |  |  | 20 mer | TATATACTTTAGATTGATTT |
|  | 3’-FLAP | |  |  |  |  |
|  |  | FITC |  |  | 49 mer | AGCTACCATGCCTGCACGAATTAAGCAATTCGTAATCATGGTCATAGCT |
|  |  |  |  |  | 47 mer | AGCTATGACCATGATTACGAATTGCTTGGAATCCTGACGAACTGTAG |
|  |  |  |  |  | 22 mer | AATTCGTGCAGGCATGGTAGCT |
|  | 3’-OH |  |  |  |  |  |
|  |  |  |  |  | 22 mer | AATTCGTGCAGGCATGGTAGCT |
|  |  | FITC |  |  | 49 mer | AGCTACCATGCCTGCACGAATTAAGCAATTCGTAATCATGGTCATAGCT |
|  | 5’-OH |  |  |  |  |  |
|  |  |  |  | FITC | 49 mer | AGCTACCATGCCTGCACGAATTAAGCAATTCGTAATCATGGTCATAGCT |
|  |  |  |  |  | 22 mer | AGCTATGACCATGATTACGAAT |
|  | RVFssDNA |  |  |  |  |  |
|  | *Fork (0ntRVF)* | |  | Cy3 | 31 mer | GACGCTGCCGAATTCTACCAGTGCCTTGCTA |
|  | *5ntRVF* |  |  | Cy3 | 36 mer | GACGCTGCCGAATTCTACCAGTGCCTTGCTATTTTT |
|  | *10ntRVF* |  |  | Cy3 | 46 mer | GACGCTGCCGAATTCTACCAGTGCCTTGCTATTTTTTTTTT |
|  | *20ntRVF* |  |  | Cy3 | 56 mer | GACGCTGCCGAATTCTACCAGTGCCTTGCTATTTTTTTTTTTTTTTTTTTTTTTTT |
|  | *25ntRVF* |  |  | Cy3 | 61 mer | GACGCTGCCGAATTCTACCAGTGCCTTGCTATTTTTTTTTTTTTTTTTTTTTTTTT  TTTTT |
|  | *RVFcomp1 ** |  |  |  | 30 mer | ATCTGTTGTAATCGTCAAGCTTTATGCCGTT |
|  | *RVFcomp 2 ** |  |  |  | 61mer | GAACGGCATAAAGCTTGACGATTACAACAGATCATGGAGCTGTCTAGAGGAT  CCGACTATCGA |
|  | *RVFcomp 3 ** |  |  |  | 60 mer | ATCGATAGTCGGATCCTCTAGACAGCTCCATGTAGCAAGGCACTGGTAGAAT  TCGGCAGCGT |
| Stopped-Flow | |  |  |  |  |  |
|  |  | Cy3 |  |  | 43 mer | TTTTTTTTTTTTTTTTTTTTTTTTTTTTTTTTTTTTTTTTTTT |
|  |  |  |  | Cy3 | 43 mer | TTTTTTTTTTTTTTTTTTTTTTTTTTTTTTTTTTTTTTTTTTT |
| Bio-Layer interferometry | | |  |  |  |  |
|  | dT ssDNA | |  |  |  |  |
|  |  | Biotinylated | |  | 9 mer | TTTTTTTTT |
|  |  | Biotinylated | |  | 10 mer | TTTTTTTTTT |
|  |  | Biotinylated | |  | 15 mer | TTTTTTTTTTTTTTT |
|  |  | Biotinylated | |  | 18 mer | TTTTTTTTTTTTTTTTTT |
|  |  | Biotinylated | |  | 20 mer | TTTTTTTTTTTTTTTTTTTT |
|  |  | Biotinylated | |  | 23 mer | TTTTTTTTTTTTTTTTTTTTTTT |
|  |  | Biotinylated | |  | 25 mer | TTTTTTTTTTTTTTTTTTTTTTTTT |
|  |  | Biotinylated | |  | 33 mer | TTTTTTTTTTTTTTTTTTTTTTTTTTTTTTTT |
|  |  | Biotinylated | |  | 40 mer | TTTTTTTTTTTTTTTTTTTTTTTTTTTTTTTTTTTTTTTT |
|  |  | Biotinylated | |  | 43 mer | TTTTTTTTTTTTTTTTTTTTTTTTTTTTTTTTTTTTTTTTTTT |
|  | RVF25ssDNA |  | |  |  |  |
|  |  |  | | Cy3 | 61 mer | GACGCTGCCGAATTCTACCAGTGCCTTGCTATTTTTTTTTTTTTTTTTTTTTTTTT  TTTTT |
|  |  |  | |  | 30 mer | ATCTGTTGTAATCGTCAAGCTTTATGCCGTT |
|  |  |  | |  | 61mer | GAACGGCATAAAGCTTGACGATTACAACAGATCATGGAGCTGTCTAGAGGAT  CCGACTATCGA |
|  |  | Biotinylated | |  | 60 mer | ATCGATAGTCGGATCCTCTAGACAGCTCCATGTAGCAAGGCACTGGTAGAAT  TCGGCAGCGT |
| smFRET analysis | |  | |  |  |  |
|  |  | Cy5 labeled  Z position | |  | 88 mer | CCCTACATCCATTCCTCGCGTTTTTTT(Z)TTTTTTTTTTTT TTTTTTTTTTTTTTTTTTTTTTTTTTTTTTTTTTTTTTTTTTTTTTTTT |
|  | Cy3 |  | | Biotinylated | 20 mer | CGCGAGGAATGGATGTAGGG |

**Supplementary Table 1:** Oligonucleotides used in this study. * These oligonucleotides are combined with either of Fork (0ntRVF), 5ntRVF, 10ntRVF, 20ntRVF, 25ntRVF to form reversed fork substrates.

**REFERENCES**

1. Johnson, K.A., Simpson, Z.B., and Blom, T. (2009). Global kinetic explorer: a new computer program for dynamic simulation and fitting of kinetic data. Anal Biochem *387*, 20–29. https://doi.org/10.1016/j.ab.2008.12.024.

2. Johnson, K.A. (2023). History of advances in enzyme kinetic methods: From minutes to milliseconds. Enzymes *54*, 107–134. https://doi.org/10.1016/bs.enz.2023.07.005.

3. Senavirathne, G., Jaszczur, M., Auerbach, P.A., Upton, T.G., Chelico, L., Goodman, M.F., and Rueda, D. (2012). Single-stranded DNA scanning and deamination by APOBEC3G cytidine deaminase at single molecule resolution. J Biol Chem *287*, 15826–15835. https://doi.org/10.1074/jbc.M112.342790.

4. Taylor, M.R.G., Špírek, M., Chaurasiya, K.R., Ward, J.D., Carzaniga, R., Yu, X., Egelman, E.H., Collinson, L.M., Rueda, D., Krejci, L., et al. (2015). Rad51 Paralogs Remodel Pre-synaptic Rad51 Filaments to Stimulate Homologous Recombination. Cell *162*, 271–286. https://doi.org/10.1016/j.cell.2015.06.015.

5. Verma, A.R., Ray, K.K., Bodick, M., Kinz-Thompson, C.D., and Gonzalez, R.L. (2024). Increasing the accuracy of single-molecule data analysis using tMAVEN. Biophys J, S0006-3495(24)00038-9. https://doi.org/10.1016/j.bpj.2024.01.022.

6. Thompson, C.D.K., Sharma, A.K., Frank, J., Gonzalez, R.L., and Chowdhury, D. (2015). Quantitative Connection between Ensemble Thermodynamics and Single-Molecule Kinetics: A Case Study Using Cryogenic Electron Microscopy and Single-Molecule Fluorescence Resonance Energy Transfer Investigations of the Ribosome. J Phys Chem B *119*, 10888–10901. https://doi.org/10.1021/jp5128805.

**SUPPLEMTARY FIGURES**

**Supplementary Figure S1: Characterization of BCDX2 complex and RAD51 protein.**

(**A**) Schematic representation of BCDX2 complex and analysis of purified protein used in this study, including RAD51, BCDX2, BC and DX2 proteins (5 µg each), by SDS-PAGE.

(**B**) Mass spectrometry analysis of the upper bands of BCDX2 complex in panel A identifies RAD51B and RAD51C components. Mass spectrometry analysis of the lower bands of BCDX2 complex in panel A identifies RAD51D and XRCC2 components.

(**C**) Gel filtration analysis of the BCDX2 (50 µL of 10 µM) complex using Superdex200 Increase 5/150 GL column. Coomassie-stained SDS-PAGE gel of the indicated fractions (Upper). The analysis of protein bands was assessed using ImageQuant TL (Bottom).

(**D**) Mass photometry analysis of BCDX2 complex (25 nM).

(**E**) Gel filtration analysis of the RAD51 protein (100 µL of 2 µM) using Superdex200 Increase 10/300 GL column. Western-blot analysis was performed using rabbit anti-RAD51 (BioAcademia, 70-002) of the indicated fractions (Upper). The chemiluminescent signals were assessed using Fujifilm MultiGauge software (Bottom).

(**F**) and (**G**) Representative electron microscopy image of RAD51 (5 μM) in the presence of Phi X 174 circular ssDNA, ATP and magnesium ions followed by incubation for either 10 minutes (**F**) or 6 hours (**G**). Samples were stained with uranyl acetate. Zoomed area depicts RAD51 ring structure. Representative oligomeric rings are depicted with yellow circles. Scale bar represents 100 nm.


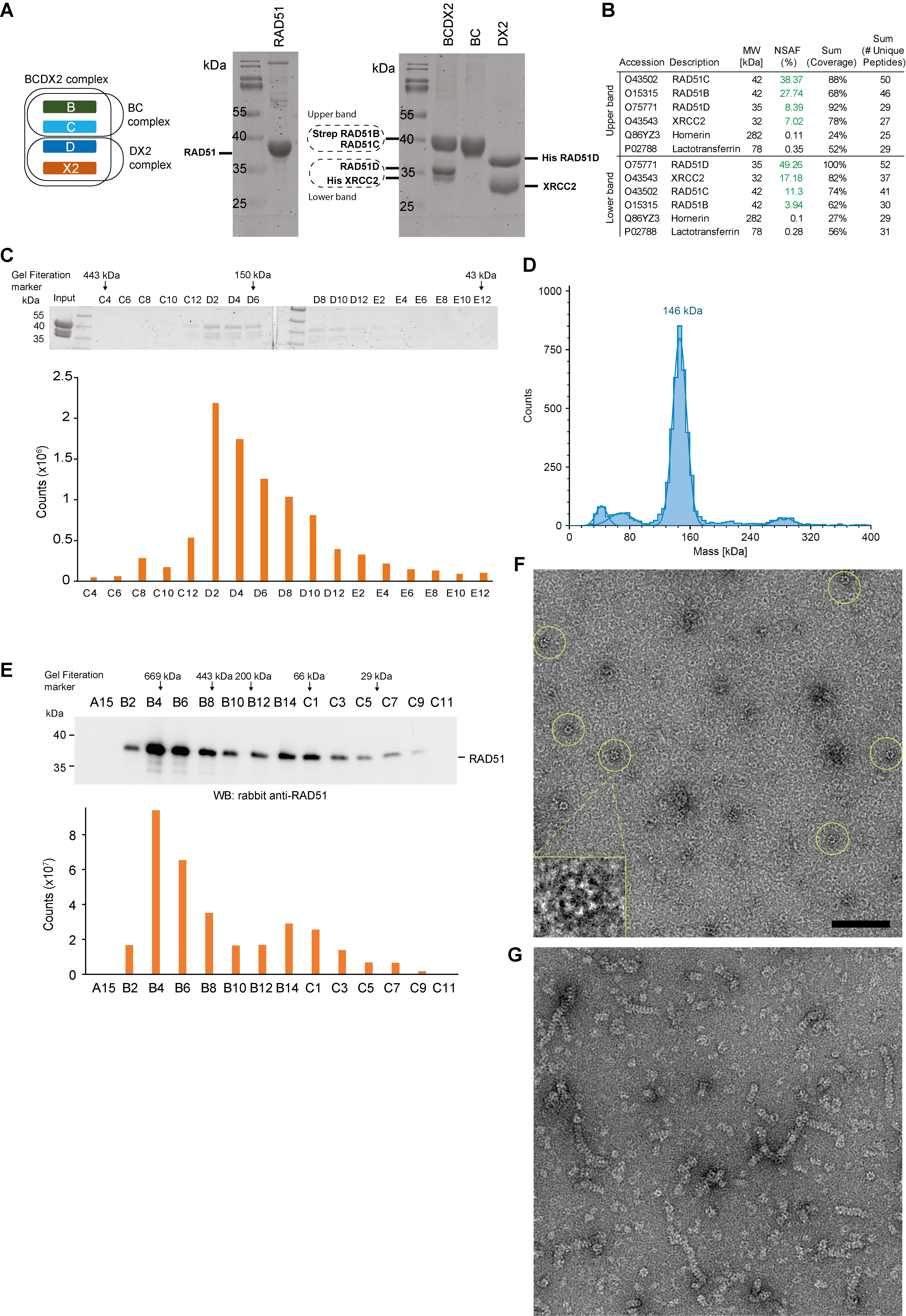


**Supplementary Figure S2: EM analysis of BCDX2 complex.**

(**A**) 3D reconstruction of BCDX2 complex using negative staining and electron microscopy. Overlay of the density map with AlphaFold prediction of the BCDX2 complex. Scale bar = 5 nm.

(**B**) FSC curve from negative stain EM data analysis.

(**C**) 2D class averages of BCDX2 in the absence of DNA, and presence of ssDNA or reversed replication fork (RVF1) using negative staining electron microscopy. Bar represents 10 nm.

(**D**) Representative images of Ni-NTA-nanogold particles bound to N-terminal His-tag of XRCC2.


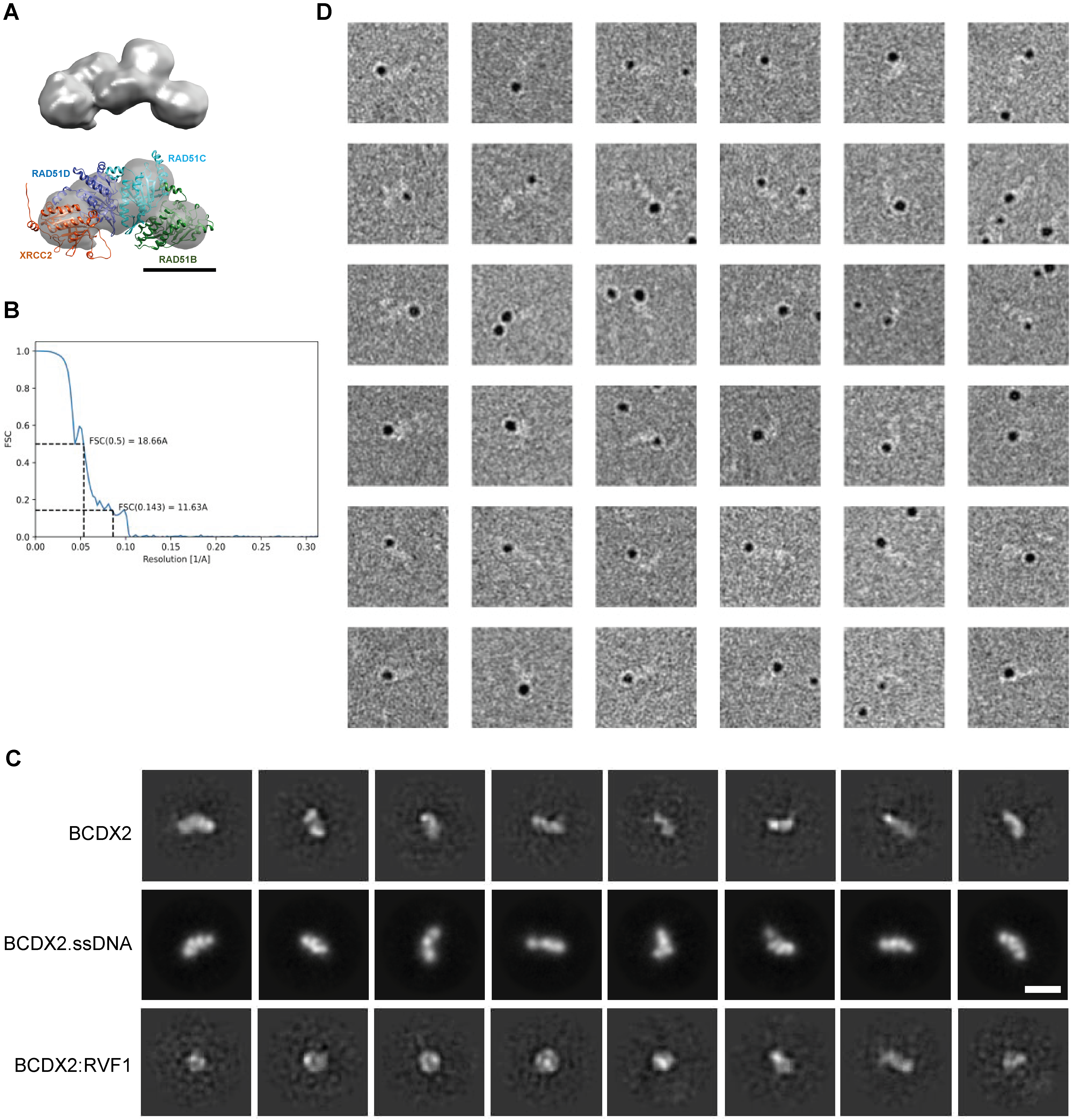


**Supplementary Figure S3: Characterization of BCDX2 DNA binding.**

(**A**) Representative EMSA images showing BCDX2, BC, and DX2 complexes (100, 200 or 400 nM) binding to 20 nM FITC-labelled ssDNA, dsDNA, reversed replication fork containing ssDNA overhang (RVF1) and fully reversed fork-like substrate (RVF2). Following the incubation, protein-DNA complexes were crosslinked and resolved in an agarose gel.

(**B**) EMSA of BCDX2, BC, and DX2 complexes (100, 200 or 400 nM) binding to 20 nM FITC-labelled Y form and 5’overhang DNA. Following the incubation, protein-DNA complexes were crosslinked and resolved in an agarose gel (Left). Percentage of DNA binding by BCDX2, BC, and DX2 complexes to Y fork and overhang DNA substrates. n=3 independent experiments; data are means s.d. (Right)

(**C**) Representative EMSA images showing BCDX2 and DX2 complexes (100, 200 or 400 nM) binding to 20 nM FITC-labelled reversed replication fork containing ssDNA overhang (RVF1), 3’flap, 3’overhang and 27mer ssDNA substrates. Following the incubation, protein-DNA complexes were crosslinked and resolved in an agarose gel.

(**D**) EMSA of BCDX2 complex (100, 200 or 400 nM) binding to 20 nM FITC-labelled reversed replication fork containing ssDNA overhang (RVF1) or dsDNA containing ssDNA gap (GAP) substrates. Following the incubation, protein-DNA complexes were crosslinked and resolved in an agarose gel (Uper). Percentage of DNA binding by BCDX2 to DNA substrates. n=3 independent experiments; data are means s.d. (Bottom).


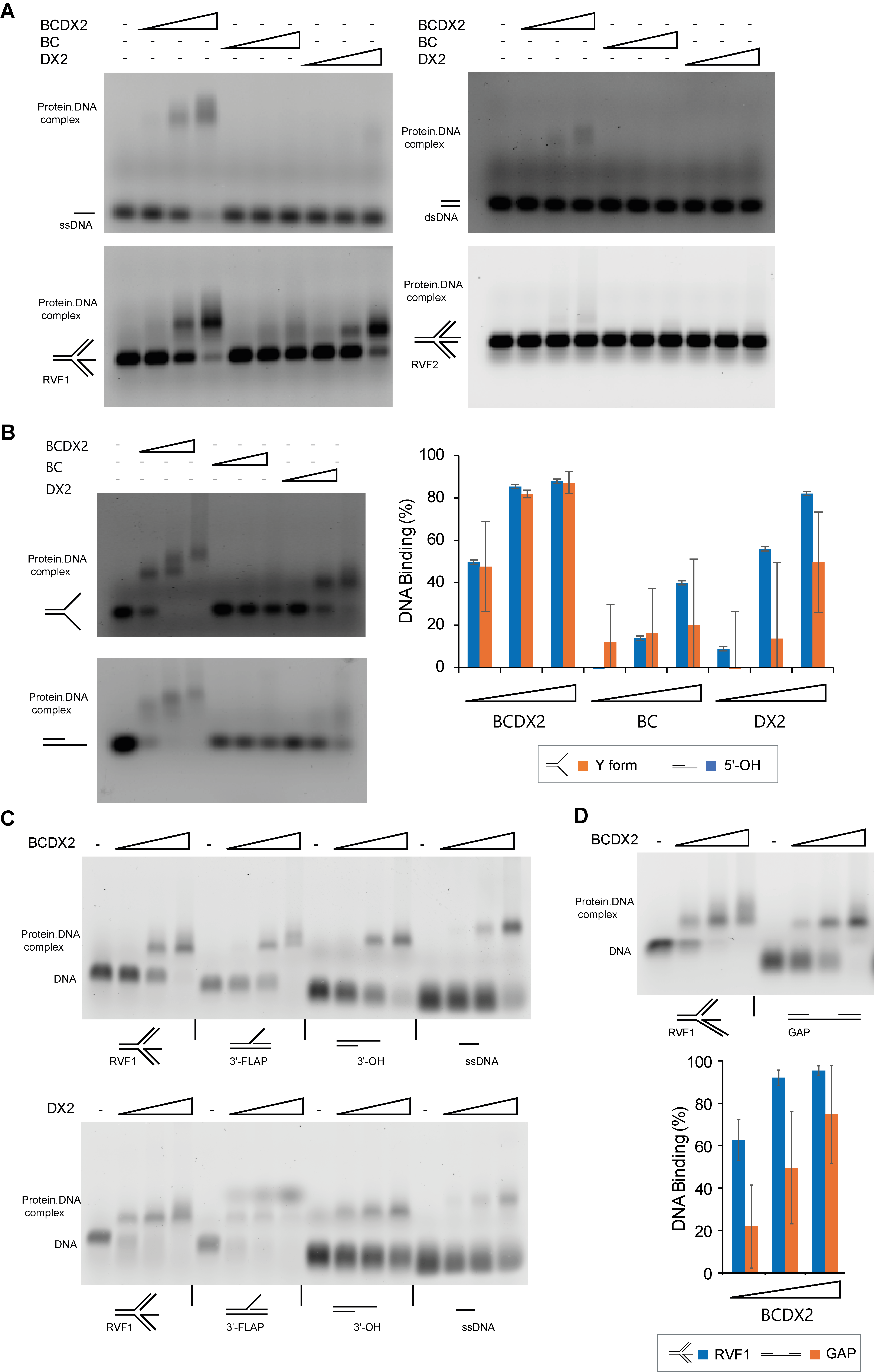


**Supplementary Figure S4: Characterization of mutant BCDX2 complexes.**

**(A**) Charts representing DNA binding of BCDX2 complex containing WT or mutant variants of DX2 (D(R266A)X2 and DX2(R159A)) using Bio-layer interferometry (BLI). Increasing concentrations of BCDX2 (12.5, 25, 50 and 100 nM) were bound to 15 nM 5’-biotinylated ssDNA conjugated to streptavidin-loaded biosensors. The left panel represents the association phase, and the right panel shows dissociation phase facilitated by addition of 100-fold excess unlabeled DNA (time point = 240 sec).

(**B**) Representative EMSA image of DX2 complex (100, 200, and 400 nM) containing WT or mutant variants of DX2 (D(R266A)X2 and DX2(R159A)) binding to 20 nM FITC-labelled reversed replication fork containing ssDNA overhang (RVF1). Following the incubation, protein-DNA complexes were crosslinked and resolved in an agarose gel.

**
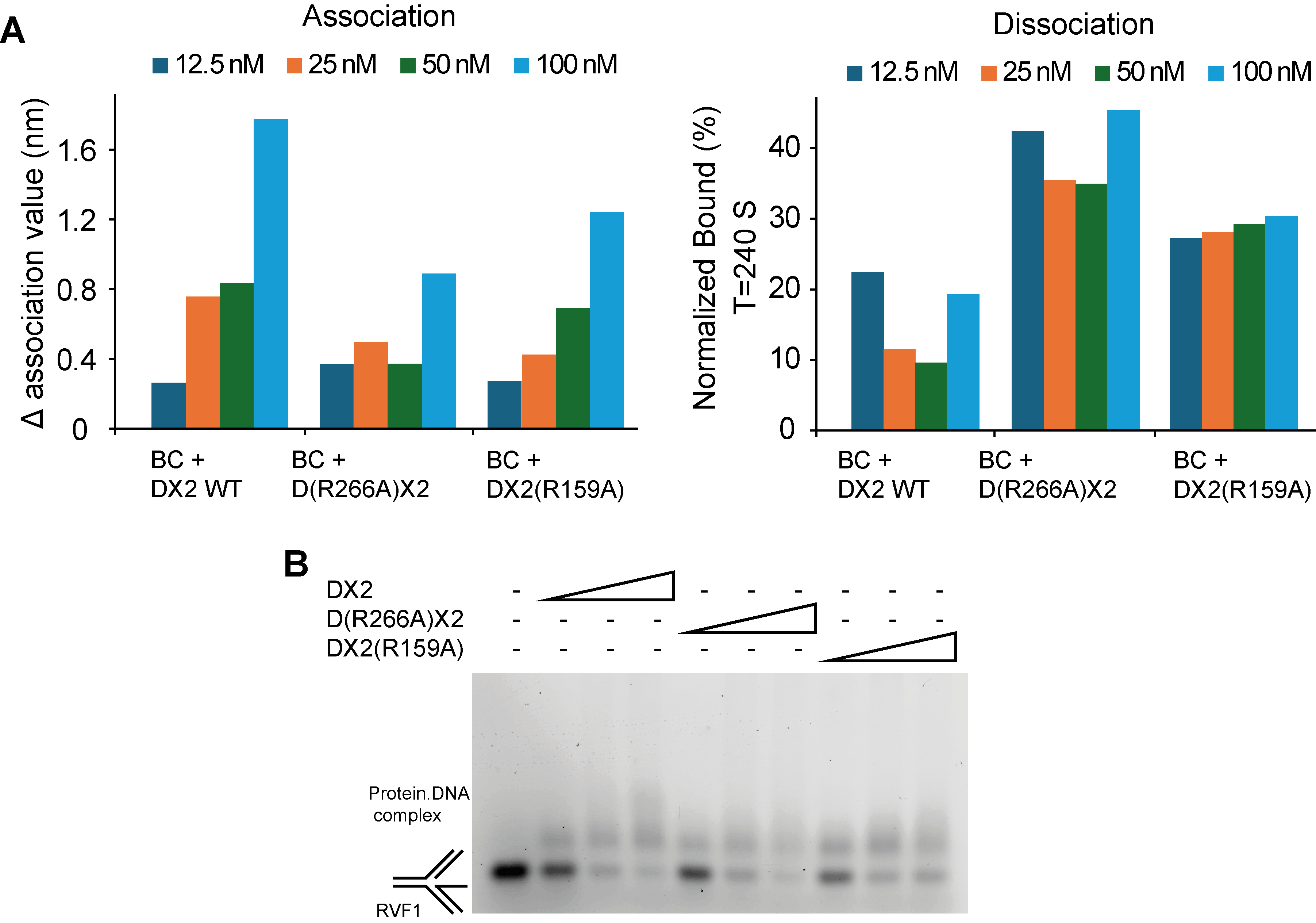
**

**Supplementary Figure S5: Kinetic analysis of ssDNA interaction with BCDX2.**

(**A**) The kinetic model of ssDNA interaction with BCDX2.

(**B** and **C**) Two independent biological replicates of the stopped-flow kinetic experiment. (Left) Fluorescence traces (excitation 545 nm, emission > 550 nm) recorded upon mixing 30 nM ssDNA (Cy3-43mer) with BCDX2 (20, 40, 60, 80, 100, 120, 140, 160, 180, 200, 300 or 400 nM). (Right) Kinetic parameters and scaling factors obtained by global fitting the kinetic data (B or C) using numerical integration of rate equations derived from the kinetic model shown in (A). Each stopped-flow trace represents an average of 3 to 4 replicates. The solid lines represent the best global fit to the kinetic data. Standard errors (± s.e.) was calculated from the covariance matrix during nonlinear regression. All experiments were performed in SF buffer at 37°C.


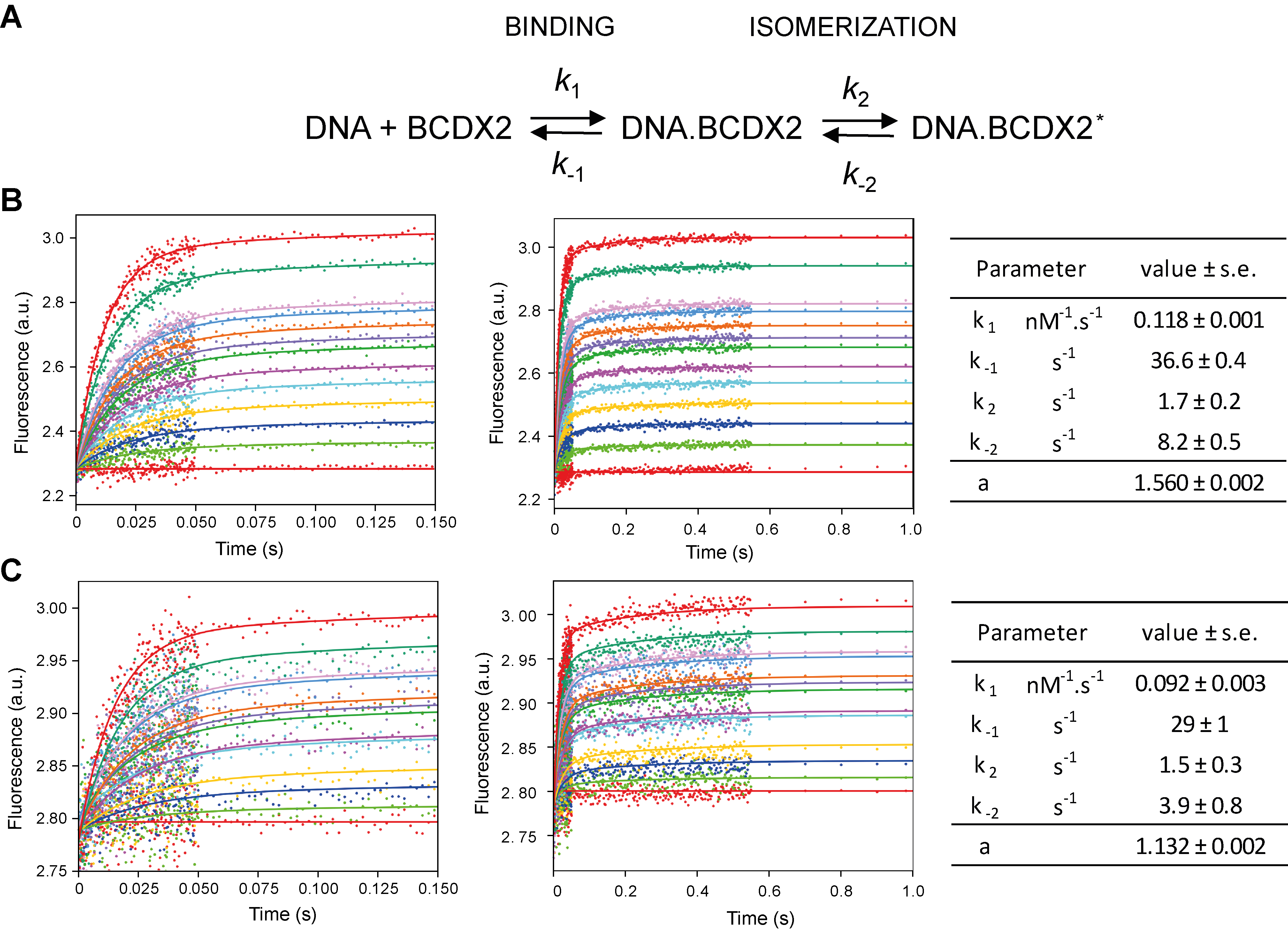


**Supplementary Figure S6: Kinetic analysis of ssDNA interaction with RAD51.**

(**A**) The kinetic model of ssDNA interaction with RAD51.

(**B** and **C**) Two independent biological replicates of the stopped-flow kinetic experiment. (Left) Fluorescence traces (excitation 545 nm, emission > 550 nm) recorded upon mixing 30 nM ssDNA (Cy3-43mer) with RAD51 (25, 50, 100, 200, 300, 400, 500, 750, 1000, 1500 or 2000 nM). (Right) Kinetic parameters and scaling factors obtained by global fitting of the kinetic data from (B) or (C) using numerical integration of rate equations derived from the kinetic model shown in (A). Each stopped-flow trace represents an average of 3 to 4 replicates. The solid lines represent the best global fit to the kinetic data. Standard errors (± s.e.) was calculated from the covariance matrix during nonlinear regression. All experiments were performed in SF buffer at 37°C.


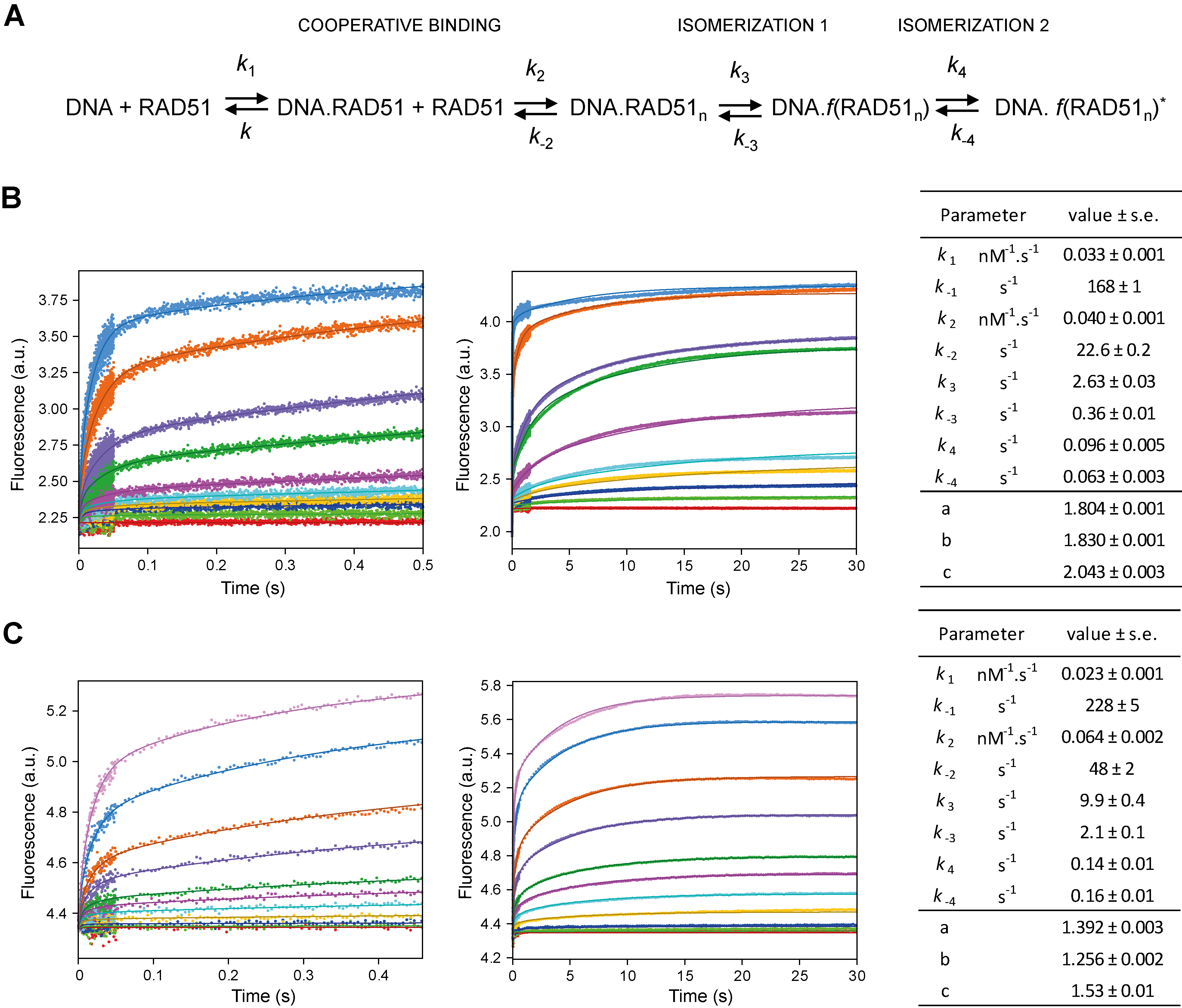


**Supplementary Figure S7: Conventional fitting of kinetic data.**

(**A**) The fluorescence traces obtained upon mixing BCDX2 (20, 40, 60, 80, 100, 120, 140, 160, 180, 200, 300 or 400 nM) with 30 nM ssDNA (Cy3-43mer) fit a double exponential function, solid line (Equation 1) (left). The concentration dependence of the observed rates from the fast and slow phases derived by fitting data in A with error bars showing the standard errors (middle). The concentration dependence of the amplitude of the fast phase (*A*_1_), slow phases (*A*_2_) and total amplitude (*A*_TOT_). The solid lines represent the best fit to the hyperbola (right).

(**B**) The fluorescence traces obtained upon mixing RAD51 (25, 50, 100, 200, 300, 400, 500, 750, 1000, 1500 or 2000 nM) with 30 nM ssDNA (Cy3-43mer) fit a triple exponential function (left). The concentration dependence of the rates of the three observed phases (middle, *k*_obs1_, *k*_obs2_ and *k*_obs3_). The concentration dependence of the amplitude of the three observed phases (right*, A*_1_, *A*_2_ and *A*_3_,). The solid lines represent the best fit, and the error bars show the standard errors.

(**C**) Same as B except for RAD51 K133R mutant.

(**D**) Same as B except for RAD51 K133A mutant.


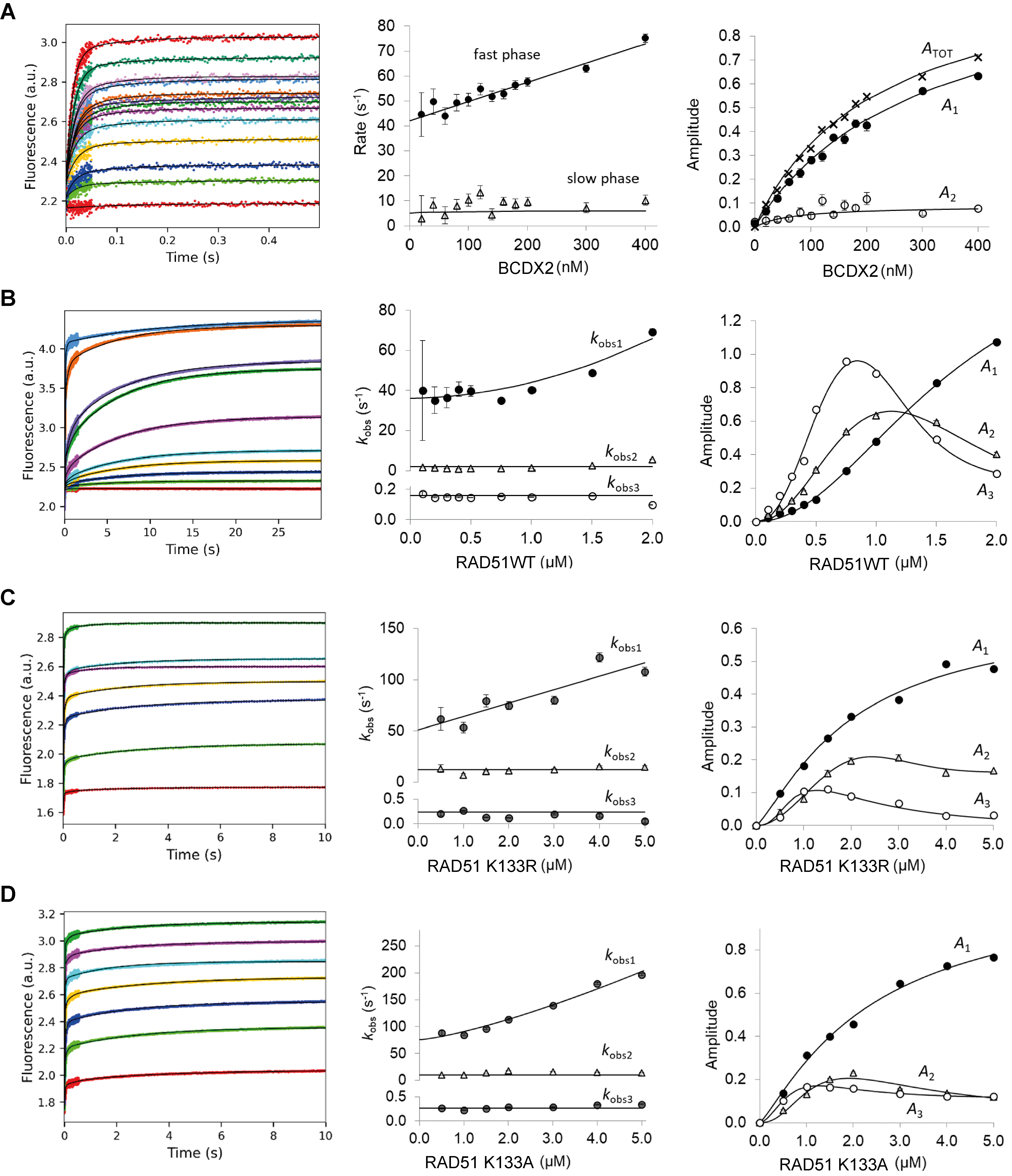


**Supplementary Figure S8: RAD51 interaction with BCDX2 and kinetic analysis of DNA interaction with BCDX2 and RAD51.**

(**A**) Pull-down assay with purified RAD51 and BCDX2 using Strep-Tactin beads. Western blot analyses were performed with anti-XRCC2 and RAD51 antibodies.

(**B**) The kinetic model illustrating the simultaneous interaction of BCDX2 and RAD51 with DNA. Stopped-flow fluorescence traces (Left - rapid binding phase; Right - slow filament formation) upon mixing 30 nM DNA with:

(**C**) 0 – 500 nM RAD51,

(**D**) 0 – 500 nM RAD51 at presence of 25 nM BCDX2,

(**E**) 0 – 500 nM RAD51 at presence of 50 nM BCDX2,

(**F**) 0 – 500 nM RAD51 (the replicate experiment),

(**G**) 0 – 500 nM RAD51 at presence of 100 nM BCDX2,

(**H**) 0 – 200 nM BCDX2,

(**I**) 0 – 200 nM BCDX2 at presence of 200 nM RAD51, and

(**J**) 0 – 200 nM BCDX2 at presence of 300 nM RAD51. Each stopped-flow trace represents an average of 3 to 4 replicates. The solid lines represent the best global fit to the kinetic data.

(**K**) Rate constants for individual steps determined by simultaneous fitting of the kinetic data from (B) to (J). Different scaling factors *f* were used for individual data sets reflecting variations in detector sensitivity. The factors a = 1.65 ± 0.03, b = 1.16 ± 0.01, c = 1.23 ± 0.01, and d = 1.23 ± 0.01, defining relative change in fluorescence of individual reaction species, were determined globally and applied to all experiments. The standard error (± s.e.) was calculated from the covariance matrix during nonlinear regression. All experiments were performed in SF buffer at 37°C.


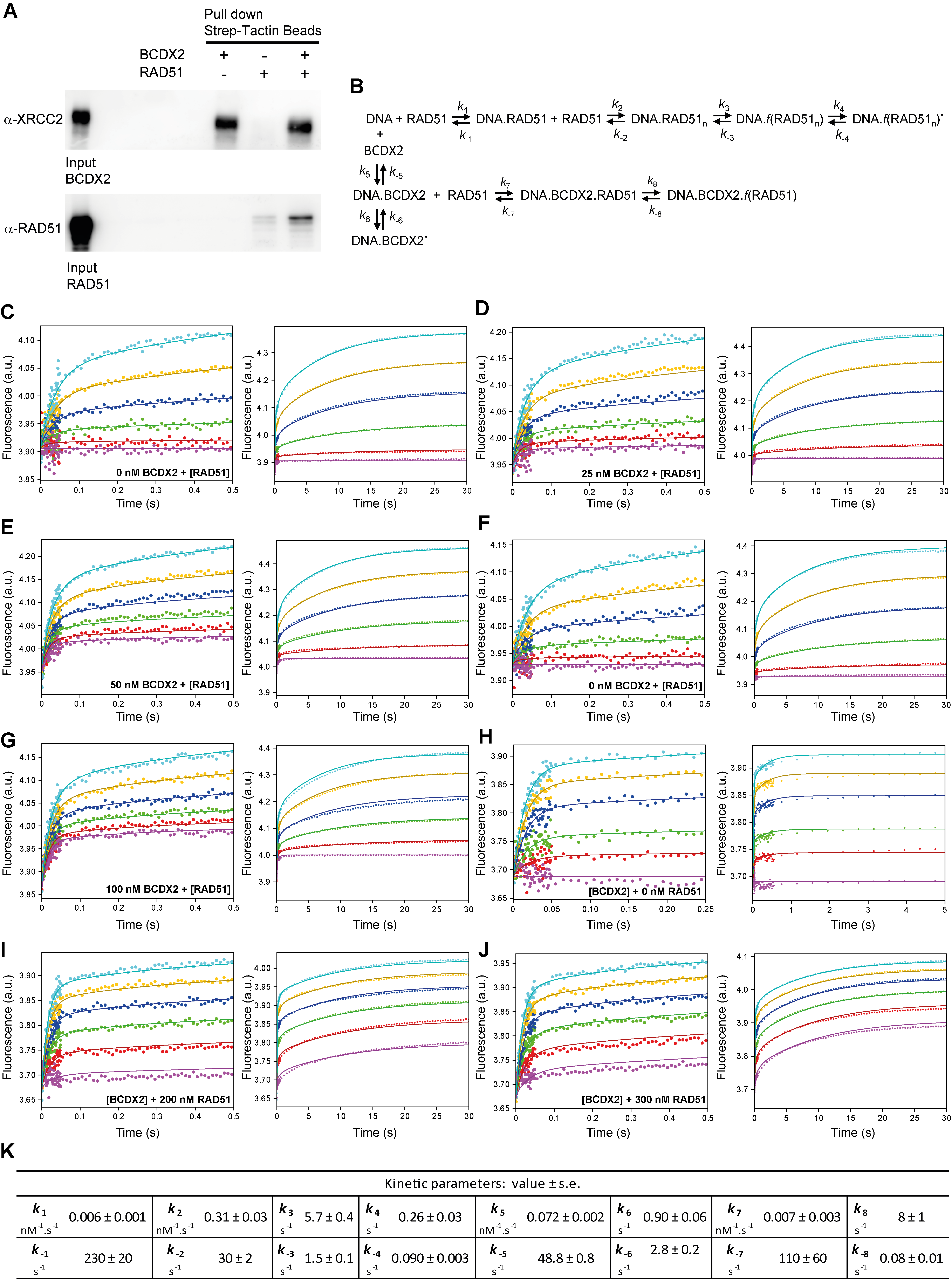


**Supplementary Figure S9: Reaction coordinates for DNA interaction with RAD51 and BCDX2.**

Free energy profiles were constructed based upon the kinetic data using the Eyring equation at reference temperature 310 K and reference concentration 1 μM RAD51 and 400 nM BCDX2.

(**A**) The reaction coordinates for BCDX2 interaction with ssDNA (green pathway).

(**B**) Nucleoprotein filaments formation with RAD51 alone (orange pathway).

(**C**) BCDX2-mediated nucleoprotein filaments formation (grey pathway).

The solid lines represent the individual reaction of RAD51 or BCDX2 with DNA, the dashed lines represent the results obtained during analysis of the simultaneous action of BCDX and RAD51.

**
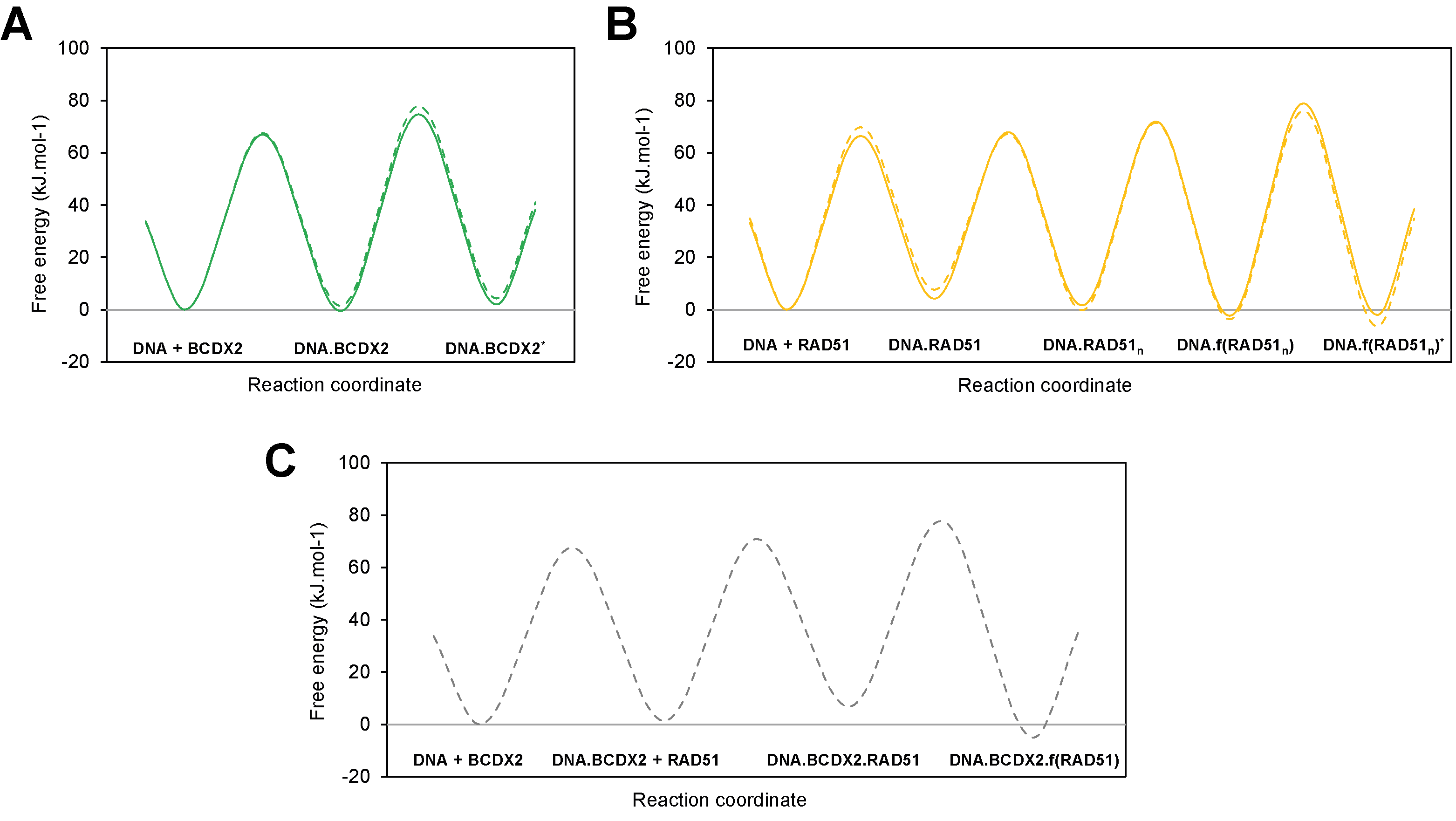
**

**Supplementary Figure S10: Comparison of BCDX2 and miniBRCA2 ssDNA binding.**

(**A**) Representative EMSA images of BCDX2 (100, 200 or 400 nM) binding to 20 nM CY3 labeled reversed replication fork substrates containing varying length of ssDNA overhang (20, 10, 5, and 0nt RVF). Following the incubation, protein-DNA complexes were crosslinked and resolved in an agarose gel.

(**B**) Quantification of BCDX2 binding to replication fork substrates containing varying length of ssDNA overhang from panel E. n=3 independent experiments; data are means s.d.

(**C** and **D**) Comparison of BCDX2 (C) and miniBRCA2 (D) binding to ssDNA of various lengths using BLI assay. BLI sensorgrams obtained using 15 nM 5’-biotinylated ssDNA of various lengths (9 to 43 nucleotides) conjugated to streptavidin-loaded biosensors. A solution of 400 nM BCDX (**C**) or miniBRCA2 (**D**) was loaded onto the biosensor and association was monitored. n=3 independent experiments; data are presented as means s.d.

(**E**) Quantification of binding amplitudes of BCDX2 or miniBRCA2 (400 nM) to ssDNA of various lengths using BLI assay from (C) and (D). n=3 independent experiments; data are presented as means s.d.


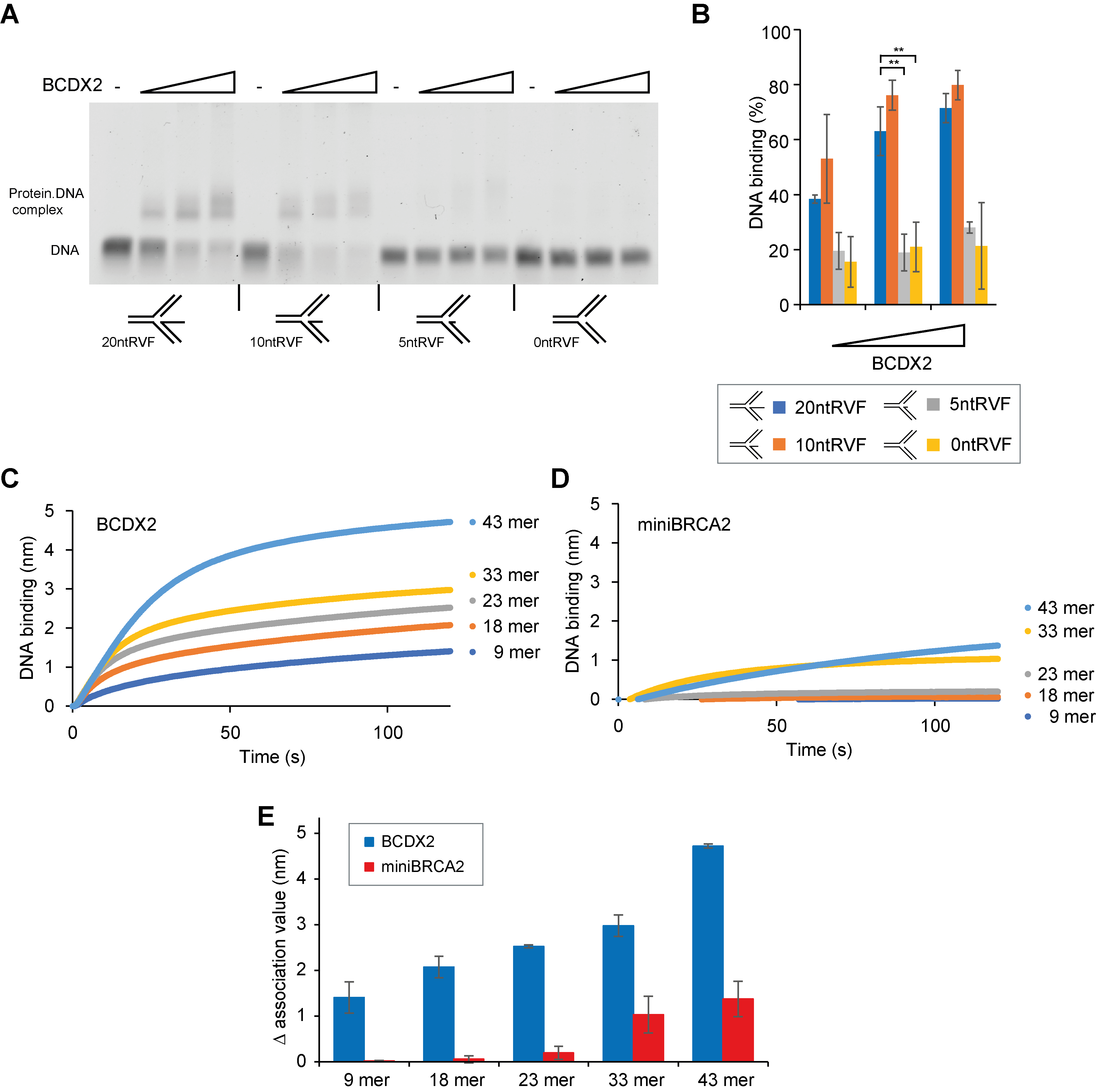

Supplement: gkae770_Supplemental_File [file gkae770_supplemental_file.docx]
